# MolEpidPred: a novel computational tool for the molecular epidemiology of foot-and-mouth disease virus using VP1 nucleotide sequence data

**DOI:** 10.1093/bfgp/elaf001

**Published:** 2025-03-05

**Authors:** Samarendra Das, Utkal Nayak, Soumen Pal, Saravanan Subramaniam

**Affiliations:** Biostatistics and Bioinformatics Facility, ICAR-National Institute on Foot and Mouth Disease, Arugul, Bhubaneswar 752050, India; Biostatistics and Bioinformatics Facility, ICAR-National Institute on Foot and Mouth Disease, Arugul, Bhubaneswar 752050, India; Division of Computer Applications, ICAR-Indian Agricultural Statistics Research Institute, Pusa, New Delhi 110012, India; Biostatistics and Bioinformatics Facility, ICAR-National Institute on Foot and Mouth Disease, Arugul, Bhubaneswar 752050, India

**Keywords:** FMD, molecular epidemiology, machine learning, web-server, nucleotide, MolEpidPred

## Abstract

Molecular epidemiology of Foot-and-mouth disease (FMD) is crucial to implement its control strategies including vaccination and containment, which primarily deals with knowing serotype, topotype, and lineage of the virus. The existing approaches including serotyping are biological in nature, which are time-consuming and risky due to live virus handling. Thus, novel computational tools are highly required for large-scale molecular epidemiology of the FMD virus. This study reported a comprehensive computational tool for FMD molecular epidemiology. Ten learning algorithms were initially evaluated on cross-validated and ten independent secondary datasets for serotype prediction using sequence-based features through accuracy, sensitivity and 14 other metrics. Next, best performing algorithms, with higher serotype predictive accuracies, were evaluated for topotype and lineage prediction using cross-validation. These algorithms are implemented in the computational tool. Then, performance of the developed approach was assessed on five independent secondary datasets, never seen before, and primary experimental data. Our cross-validated and independent evaluation of learning algorithms for serotype prediction revealed that support vector machine, random forest, XGBoost, and AdaBoost algorithms outperformed others. Then, these four algorithms were evaluated for topotype and lineage prediction, which achieved accuracy ≥96% and precision ≥95% on cross-validated data. These algorithms are implemented in the web-server (https://nifmd-bbf.icar.gov.in/MolEpidPred), which allows rapid molecular epidemiology of FMD virus. The independent validation of the MolEpidPred observed accuracies ≥98%, ≥90%, and ≥ 80% for serotype, topotype, and lineage prediction, respectively. On wet-lab data, the MolEpidPred tool provided results in fewer seconds and achieved accuracies of 100%, 100%, and 96% for serotype, topotype, and lineage prediction, respectively, when benchmarked with phylogenetic analysis. MolEpidPred tool provides an innovative platform for large-scale molecular epidemiology of FMD virus, which is crucial for tracking FMD virus infection and implementing control program.

## Introduction

Foot-and-mouth disease (FMD) is one of the significant and serious viral diseases of cloven-hoofed animals including cattle, buffalo, sheep, goat, and pig among others [1]. The estimated annual economic loss attributed to the FMD in endemic regions of the world is 6.5–21 billion USD [1, 2] (United Kingdom: 3.94 billion USD [3]; India: 3.48 billion USD [4]; China: 2.5–7 billion USD [1]; African countries: 0.83–1.12 billon USD [5]; Brazil: 0.132–0.271 billon USD [6]). For instance, 2001 FMD outbreak in the United Kingdom caused death of over 6 million animals [3], which severely impacted livelihood of small farmers. The FMD is caused by an *Aphthovirus* of the *Picornaviridae* family and has seven distinct serotypes, immune-response variants of the virus, (*i.e.* O, A, C, Asia 1, SAT1–3) in global circulation [1, 2, 7]. Within these serotypes more than 65 topotypes (geographically distinct variants) and numerous genetic lineages/groups have also been identified, which are crucial for tracking the virus evolution, genetic diversity, and transmission dynamics (Supplementary File. 1) [7]. There is no cross-protection between different serotypes due to presence of unique antigenic variation [8]. As a result, infection or vaccination with one serotype does not provide immunity against others, implying the need for serotype-specific measures. Hence, FMD molecular epidemiology deals with identifying the genetic basis of disease including variants of the virus (*i.e.* serotype, topotype and genetic lineages) [7]. This has proven to be effective in understanding the virus evolution and transmission, and consequently its control (*e.g.* vaccine strain selection) [9].

Serological tests are commonly used for serotyping the FMD virus isolates by detecting serotype-specific antibodies, which helps in vaccine virus selection [10]. These tests usually suffer from: lower sensitivity, high risk due to live virus handling, time-consuming, high volume of sample requirement, and supply-chain issues [11]. Besides, these tests cannot be used for topotype and lineage identification of the virus isolate, which is crucial for tracking virus evolution and understanding transmission dynamics. Thus, molecular biological techniques including high-throughput sequencing [12] and nucleic acid amplification test [13] have been used in the FMD epidemiology [14]. These methods work by analysing the virus genetic material, offering higher sensitivity, faster results, and ability to identify topotypes and lineages. Such techniques generate huge amount of sequence data, which are continuously deposited in public-domain databases. Further, innovative computational tools and web-based solutions for FMD molecular epidemiology, presently lacking in the literature, may be developed for quick data analysis and easy decision making. These tools would have immense applications, through predicting the pathogen variants causing the outbreak, in FMD control and management including selection of appropriate vaccination strategy and timely surveillance. Such developments might require the integration of Machine Learning (ML) algorithms in animal disease epidemiology and management. Thus, there is a need for novel computational solutions that incorporate ML algorithms for quick solutions to complex FMD molecular epidemiology to enable virus evolution tracking and stronger disease management.

The ML algorithms have recently shown tremendous utilities in human disease epidemiology including infectious diseases [15], risk factor prediction [16], patient classification [17], antigen prediction [18], vaccine candidate selection [19], virus typing [20], fungus species prediction [21], microbial community prediction [22], DNA-binding protein prediction [23], *etc.* Previous studies also indicated that ML models provided competitive or even better results in disease epidemiology than the traditional methods [24]. The reason may be attributed to flexibility nature of the ML models and requirement of fewer assumptions, typically functioning in nonparametric or semi-parametric environment [23]. Nevertheless, the application of ML in animal infectious disease management is at nascent state, which requires novel computational solutions.

The current molecular epidemiology approaches for the FMD virus including serotyping are largely biological in nature, which are time-consuming and risky. Besides, for analysing sequence data to identify genetic groups (*i.e.* topotype and lineage), skilled manpower is required. So far, no computational tool is available for molecular epidemiology of the FMD virus, though it is a serious disease posing a grave threat to the livestock sectors across the world. To address this gap, we propose a novel computational approach and a web-server called MolEpidPred, which is designed to predict the serotypes, topotypes, and lineages of FMD virus isolates using VP1 nucleotide sequence data. The developed approach employs the ML strategies in three stages utilizing sequence-based features as inputs. The performance of the developed approach is evaluated on independent multiple secondary and primary wet-lab datasets. The researchers can access to the developed approach *via* a freely available user-friendly web-server.

## Materials and methods

### Data source

In this study, we used two types of data, namely secondary data and wet-lab generated data for model building and validation, respectively. The former category of data was collected from the public domain databases (*i.e.* NCBI, WRLFMD), while for the latter category, the data generated from field samples at the ICFMD laboratory were used.

### Retrieval and processing of secondary nucleotide sequence data

NCBI GenBank database (accessed on 01/11/2023), WRLFMD database (accessed on 10/11/2023), and ICAR-NIFMD data repository (accessed on 10/11/2023) were used to retrieve and compile VP1 nucleotide sequence data of the FMD virus isolates. In all the experimental molecular epidemiological investigations, VP1 coding region of the FMD virus genome is usually sequenced as it is the most variable region in the genome and commonly used for tracing outbreak [25–28]. Nucleotide sequences encoding the capsid protein VP1 is sufficient for molecular epidemiology of the FMD virus [28, 29].

Secondary VP1 sequence data of the FMD virus isolates with annotated serotype information, available till date, were retrieved. The VP1 sequence data of 7993 virus isolates (as on 01/11/2023) from the GenBank and WRLFMD databases were collected. The sequence data of the isolates with non-nucleotide bases (*e.g. M*, *N*), missing serotype information, and with shorter lengths (<300 bp) were removed. Through this, sequence data of 7961 virus isolates (annotated with seven serotypes) were retained for further analysis (Supplementary File 2). Out of these selected isolates, 2463 isolates reported from ten FMD-endemic countries were used for independent external evaluation of trained models (Supplementary File 3). Alternatively, 5498 virus isolates reported across all the FMD-endemic countries were used for model building (training and internal validation). Further, topotype and lineage information of the retrieved virus isolates of each serotype were collected from the WRLFMD database, ICAR-NIFMD data repository, and published literature [25–38]. Besides, wet-lab generated VP1 sequence data of 74 FMD virus isolates collected from various outbreak areas in India and sequenced at our ICFMD lab facility were used in this study. The detailed description of sample collection and sequencing is given in Supplementary File 4.

### Generation of k-mer features

Biological sequences are strings and cannot be directly used for predictive model building. Thus, they are converted into numerical feature vectors for model training. For this purpose, the sequence-based features have been proven effective in a number of prediction tasks due to their easiness in implementation [18–21, 23]. Specifically, the *k-mer* based feature descriptors have been utilized in several virus bioinformatics applications [11, 20]. The *k-mer* features consist of all possible contiguous sub-sequences of length *k* derived from the VP1 nucleotide sequences, briefly described in Supplementary File 5. For instance, the nucleotide sequences would yield a total of 64 3-mers, namely "AAA", "AAT", ‘AAG’, *etc.*, listed in Supplementary File 6. To implement the *k-mer* technique for obtaining feature count vectors, we executed the *kmer* R package. Another challenge in *k-mer* feature generation is the determination of optimal *k*, as higher value of *k* increases computational time multi-fold. Thus, an empirical approach, given in Supplementary File 5, was used for determining the optimal *k* to generate the optimal feature set.

### Data balancing

The retrieved pre-training dataset had unequal number of isolates in each serotype category (*e.g.* O: 4209; A: 1853; Asia 1: 613, *etc.*). Imbalanced data is a wide concern for global FMD epidemiology modelling; as O serotype is the most prevalent serotype causing 92% outbreaks compared to others [39]. In order to avoid prediction bias toward the O serotype having a larger number of virus isolates, a balanced dataset with an equal number of isolates from all the seven serotypes was taken in to account. In this context, down-sampling, up-sampling, and synthetic minority over sampling technique (SMOTE) [40] sampling of the training data are commonly practiced. So, we used SMOTE technique to balance the pre-training dataset, so that each serotype will have equal representation in model training and testing.

### Serotype prediction with machine learning algorithms

We utilized seven ML methods such as support vector machine (SVM) [41], random forest (RFF) [42], adaptive boosting (ADB) [23], extreme gradient boosting (XGB) [23], gradient boosting machine (GBM) [43], artificial neural network (ANN) [11], and decision tree (TRE) [11]. Besides, three statistical methods such as *K-th* nearest neighbour (KNN) [11], multiclass logistic regression (MLR), [11] and naïve Bayes (NBS) [11] were also employed. The R-packages, namely, *e1071, randomForest, xgboost, rbooster, gbm, naivebayes, neuralnet, nnet, rpart*, and *class* were utilized to implement the SVM, RFF, XGB, ADB, GBM, NBS, ANN, MLR, TRE, and KNN methods, respectively. The learning algorithms and R packages are briefly described in Supplementary File 7 with appropriate citation, along with their tuned parameters configuration.

### Topotype and lineage prediction with machine learning algorithms

We narrowed down the top-performed algorithms based on their serotype prediction performance and further used them to predict the topotype and lineage. It’s mainly due to topotype and lineage prediction performance of an algorithm mainly depends on its serotype prediction performance. Alternatively, top-performed learning algorithms were utilized to predict the topotype and lineage of the virus isolates in the second and third steps respectively.

### Cross validation and performance metrics

The performance of the learning algorithms was evaluated through a repeated fivefold cross-validation (CV) technique, where the experiment was repeated 100 times to ensure robust results. To carry out the CV [44], the balanced dataset was randomly divided into five sub-groups of equal size and one sub-group was taken as test set and the remaining four were utilized for training. Next, the learning algorithms were trained on training set and evaluated on test set. This procedure is carried out five times, so that each sub-group gets equal representation in training and test sets. The accuracy metric was computed by averaging over the accuracy over all five test sets as well as 100 replications. The use of repeated CV allowed us to minimize overfitting and provided a more accurate estimate of model performance across different data splits.

**Figure 1:**
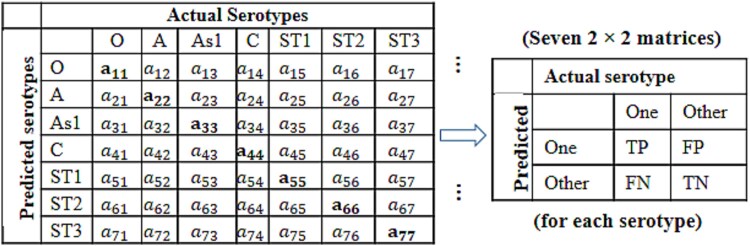
Confusion matrices for FMD virus serotype prediction. One 7 × 7 confusion matrix is decomposed into seven 2 × 2 matrices based one *versus* rest technique. As1, ST1, ST2, and ST3 are Asia 1, SAT1, SAT2, and SAT3, respectively.

We also tested performance of the learning algorithms on randomly generated test data. Here, the balanced data was randomly splitted into two parts having 70% and 30% virus isolates, respectively. Randomly chosen 70% part was considered as training set and the remaining 30% was considered as test set. The models were trained on training set and subsequently tested on the test set. For each evaluation, the results are shown in $L\times L$ confusion matrices (*e.g.*  $7\times 7$ for serotype classification) to compute classification accuracy, as shown in Fig. 1. Next, the $L\times L$ confusion matrices were decomposed into *L*  $2\times 2$ confusion matrices using one *versus* rest technique [45] (Fig. 1) to compute performance metrics, listed in Table 1. This process was repeated 500 times and then the performance metrics were determined by averaging their values over 500 replications. The higher values of performance metrics including Accuracy, Jaccard index, MCC, Precision, Recall, Sensitivity, Specificity, OP, Youden index, and F1 score indicated better performance of the algorithms and *vice-versa* (Table 1). While, the lower values of FDR, FPR, FNR, Lambda index, and runtime suggested better performance of the algorithms and *vice-versa*.

**Table 1:** Performance metrics used in evaluation of machine learning algorithms.

**Sl. No.**	**Metrics**	**Formula**
1	Accuracy	$\sum_{i=1}^7{a}_{ii}/\sum_{i, j=1}^7{a}_{ij}$
2	Bal. Accuracy	$\frac{1}{14}\sum_{i=1}^7\left({TP}_i/\left({TP}_i+{FN}_i\right)+{TN}_i/\left({TN}_i+{FP}_i\right)\right)$
3	FDR	$\frac{1}{7}\sum_{i=1}^7\left({FP}_i/\left({TP}_i+{FP}_i\right)\right)$
4	FNR	$\frac{1}{7}\sum_{i=1}^7\left({FN}_i/\left({TP}_i+{FN}_i\right)\right)$
5	FPR	$\frac{1}{7}\sum_{i=1}^7\left({FP}_i/\left({TN}_i+{FP}_i\right)\right)$
6	Jaccard index	$\frac{1}{7}\sum_{i=1}^7{TP}_i/\left({TP}_i+{FP}_i+{FN}_i\right)$
7	Lambda index	$\frac{1}{7}\sum_{i=1}^7((1-{rec}_{i})/{spec}_i)$
8	MCC	$\frac{1}{7}\sum_{i=1}^7\sqrt{\left({TP}_i{TN}_i-{FP}_i{FN}_i\right)/(\left({TP}_i+{FP}_i\right)\left({TN}_i+{FP}_i\right)\left({TP}_i+{FN}_i\right)\left({TN}_i+{FN}_i\right)})$
9	NPV	$\frac{1}{7}\sum_{i=1}^7{TN}_i/\left({TN}_i+{FN}_i\right)$
10	OP	$\frac{1}{7}\sum_{i=1}^7\left({acc}_i-|{rec}_i-{spec}_i|/\left({rec}_i+{spec}_i\right)\right)$
11	Precision	$\frac{1}{7}\sum_{i=1}^7{TP}_i/\left({TP}_i+{FP}_i\right)$
12	Recall	$\frac{1}{7}\sum_{i=1}^7{TP}_i/\left({TP}_i+{FN}_i\right)$
13	Sensitivity	$\frac{1}{7}\sum_{i=1}^7{TP}_i/\left({TP}_i+{FN}_i\right)$
14	Specificity	$\frac{1}{7}\sum_{i=1}^7{TN}_i/\left({TN}_i+{FP}_i\right)$
15	Youden index	$\frac{1}{7}\sum_{i=1}^7\left({rec}_i+{spec}_i-1\right)$
16	F1 score	$\frac{1}{7}\sum_{i=1}^7\left(2\times{prec}_i\times{rec}_i/\left({prec}_i+{rec}_i\right)\right)$

MCC: Mathew Correlation Co-efficient; NPV: Negative Prediction Value; FDR: False Discovery Rate; FNR: False Negative Rate; FPR: False Positive Rate; Bal.: Balanced; OP: Optimized precision; TP: True positive; FP: False positive; TN: True negative; FN: False negative; rec: recall; spec: specificity; prec: precision; *i* represents serotype (O, A, Asia1, C, SAT1–3)

### MolEpidPred computational approach

**Figure 2:**
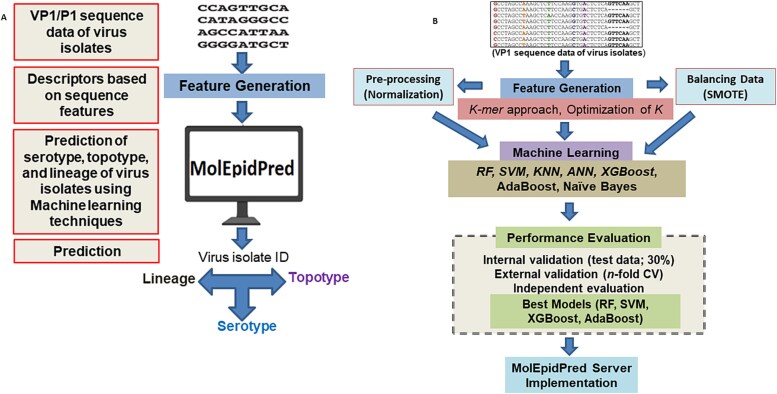
Operational framework and algorithm of the MolEpidPred computational tool. (A) Operational framework of the MolEpidPred computational solution. (B) Algorithm of the MolEpidPred computational solution.

The molecular epidemiology prediction of the FMD virus is a three-step process, step 1: prediction of serotype; step 2: prediction of topotype of virus isolates within each predicted serotype; step 3: prediction of lineage within each predicted topotype. Initially, we considered ten learning algorithms for performance analysis based on their ability to predict the serotypes of the virus isolates. Then, top-performer models were further considered for prediction of topotype and lineage of the virus isolates for each serotype. This was done to reduce the computational complexity of the proposed MolEpidPred approach*.* The operational framework and major analytical steps of the proposed computational solution are shown in Fig. 2.

### Molecular epidemiology of the field FMD virus isolates

Unlike the secondary data available in databases, the raw sequence data usually contain missing nucleotide bases at certain positions and cannot be directly taken for bioinformatics analysis. Therefore, the utility of the developed MolEpidPred computational tool was demonstrated on field virus isolates isolated from the tongue and foot epithelium samples of infected cloven-hoofed animals (*e.g.* cattle, buffalo, pig, and goat) collected from various outbreak hotspots across India during the year 2018–19. Here, the samples were collected by the state FMD research centres and state veterinary departments in India, following the world organization of animal health approved guidelines. Next, these samples were sent to our ICFMD facility, a Food and Agriculture Organization reference laboratory for the FMD, located in India for sequencing. The detailed methodology used for sequencing was given in Supplementary File 4 and described earlier by Subramaniam *et al.* [28]. Here, we considered the raw VP1 sequence data of the 74 virus isolates to study the utility of the MolEpidPred approach and also supplied at https://github.com/sam-dfmd/MolEpidPred_Data.

## Results

### Analysis of k-mer features

**Figure 3:**
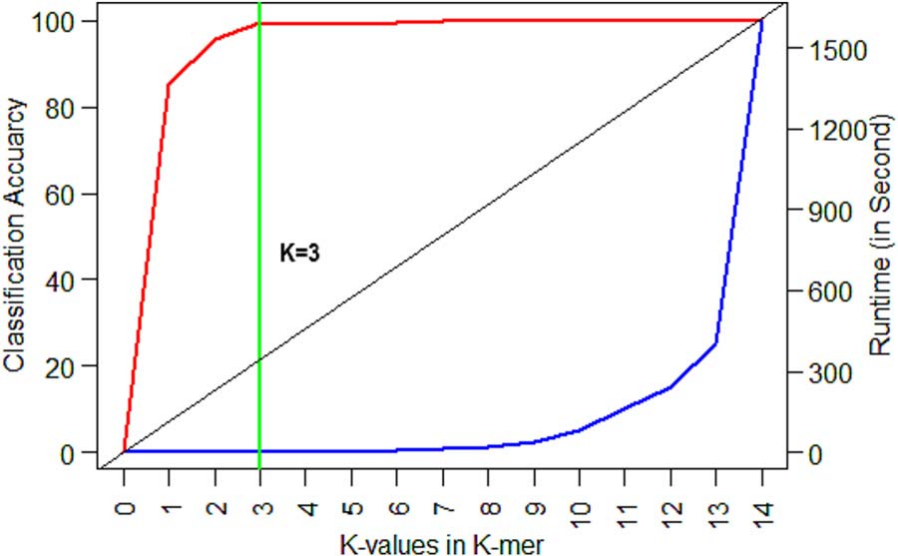
Selection of optimal *k* for *k-mer* feature generation. X-axis represents the *k*-values (0, 1, 2, …, 14). Y1-axis represents the computed predictive accuracy over five-fold cross-validation; Y2-axis represents the required runtime to execute the R-code.

The computational cost of *k-mer* based predictive model building for nucleotide sequence data depends on the value of *k* (*e.g. k* = 10, #features = 10 48 576). For optimal feature set generation, we determined the optimal value of *k* through training the SVM classifier. Here, *k* was set at 1 to 14 for generation of corresponding *k-mer* features and each *k-mer* feature set was assessed through computed classification accuracy (over five-fold CV) and required runtime. The classification accuracy was found to be increased up to *k* = 3, then it got stabilized (Fig. 3). Alternatively, the point of inflexion in the curve was achieved at *k* = 3 and the 3*-mer* feature set provided satisfactory results in terms of higher classification accuracy and lower runtime (Fig. 3). The 64 *3-mer* features, listed in Supplementary File 6, and their counts were taken into consideration for further analysis.

### Performance evaluation on test data for serotype prediction

The performance of the ten learning algorithms was assessed on random test data and performance metrics were averaged over 500 repetitions and shown in Table 2. The balanced accuracy and precision of the SVM (0.998, 0.996), RFF (0.997, 0.994), XGB (0.996, 0.995), ADB (0.994, 0.993), and GBM (0.992, 0.993) were observed higher compared to other algorithms (Table 2). Similar interpretation can be made for other metrics. Broadly, through all the metrics including Accuracy, F1, Jaccard, MCC, NPV, OP, Precision, Sensitivity, Specificity, and Youden index, the performance of the five algorithms (SVM, RFF, ADB, XGB, and GBM) was observed better over others (Table 2). Not much variation in performances of these five algorithms was observed on the test data. Similar observation could be made based on other performance metrics (*i.e.* FDR, FPR, FNR, lambda index) (Table 2). Among all the algorithms, the TRE model provided least accurate results. Further, computational runtime required for model training was higher for the RFF followed by ANN (Table 2).

**Table 2:** Performance analysis of machine learning algorithms on random test data replicated over 500 repetitions.

Method	Bal. ACC	F1	FDR	FNR	FPR	Jaccard	lambda	MCC	NPV	OP	Prec.	Recall/ Sens.	Spec.	Youden index	Time (Sec)
SVM	0.998 ±0.001	0.996 ±0.002	0.004 ±0.002	0.004 ±0.002	0.001 ±0.0001	0.993 ±0.005	0.004 ±0.002	0.996 ±0.003	0.999 ±0.0001	0.994 ±0.004	0.996 ±0.002	0.996 ±0.002	0.999 ±0.0001	0.996 ±0.003	6.67
RFF	0.997 ±0.002	0.995 ±0.003	0.003 ±0.003	0.005 ±0.003	0.003 ±0.0004	0.995 ±0.006	0.003 ±0.003	0.997 ±0.005	0.998 ±0.0002	0.995 ±0.006	0.994 ±0.004	0.994 ±0.004	0.995 ±0.0003	0.992 ±0.006	296.9
MLR	0.976 ± 0.002	0.979 ±0.004	0.003 ±0.005	0.006 ±0.005	0.003 ±0.007	0.979 ±0.008	0.006 ±0.004	0.972 ±0.005	0.973 ±0.007	0.958 ±0.008	0.974 ±0.006	0.976 ±0.005	0.973 ±0.007	0.977 ±0.005	5.66
ANN	0.921 ±0.066	0.854 ±0.109	0.144 ±0.107	0.144 ±0.108	0.033 ±0.024	0.763 ±0.146	0.076 ±0.121	0.822 ±0.132	0.967 ±0.024	0.753 ±0.167	0.856 ±0.107	0.856 ±0.108	0.967 ±0.024	0.823 ±0.132	77.00
XGB	0.996 ±0.004	0.991 ±0.007	0.006 ±0.007	0.005 ±0.006	0.001 ±0.0001	0.991 ±0.002	0.004 ±0.006	0.995 ±0.009	0.998 ±0.002	0.992 ±0.002	0.995 ±0.008	0.994 ±0.008	0.998 ±0.001	0.995 ±0.004	1.37
ADB	0.994 ±0.003	0.99 ±0.006	0.009 ±0.005	0.002 ±0.005	0.002 ±0.0008	0.991 ±0.003	0.005 ±0.002	0.992 ±0.004	0.995 ±0.001	0.993 ±0.002	0.991 ±0.005	0.991 ±0.006	0.998 ±0.0002	0.994 ±0.09	52.48
GBM	0.992 ±0.003	0.993 ±0.006	0.004 ±0.006	0.003 ±0.006	0.004 ±0.0002	0.991 ±0.007	0.002 ±0.006	0.991 ±0.007	0.996 ±0.001	0.991 ±0.002	0.993 ±0.006	0.989 ±0.007	0.999 ±0.002	0.992 ±0.008	27.42
TRE	0.895 ±0.007	0.829 ±0.008	0.161 ±0.006	0.159 ±0.007	0.031 ±0.055	0.728 ±0.008	0.165 ±0.007	0.809 ±0.007	0.969 ±0.006	0.766 ±0.009	0.829 ±0.005	0.831 ±0.007	0.959 ±0.005	0.810 ±0.008	3.50
NBS	0.926 ±0.015	0.907 ±0.035	0.106 ±0.031	0.097 ±0.021	0.076 ±0.069	0.813 ±0.056	0.071 ±0.026	0.849 ±0.035	0.947 ±0.053	0.85 ±0.044	0.897 ±0.031	0.908 ±0.033	0.968 ±0.054	0.895 ±0.027	0.70
KNN	0.979 ±0.011	0.977 ±0.021	0.032 ±0.021	0.031 ±0.02	0.005 ±0.003	0.976 ±0.004	0.031 ±0.021	0.973 ±0.023	0.951 ±0.037	0.963 ±0.032	0.978 ±0.002	0.987 ±0.002	0.973 ±0.003	0.975 ±0.002	15.56

Bal. ACC: Balanced Accuracy; FDR: False Discovery Rate; FNR: False Negative Rate; FPR: False Positive Rate; MCC: Mathew Correlation Co-efficient; NPV: Negative Prediction Value; OP: Optimized precision; Prec.: Precision; Sens.: Sensitivity; Spec.: Specificity; values are shown in $\mu \pm \sigma$ format, where, $\mu, \sigma$ are mean and std. deviation computed over 500 repeatations.

### Cross-validated performance for serotype prediction

The predictive accuracy (computed through five-fold CV) of the ten learning algorithms are shown in Supplementary File 8. The performance of nine algorithms (except TRE) was observed better with at least 90% predictive accuracy (SVM: 99.97%; RFF: 99.98%; XGB: 99.39%; ADB: 99.75%; GBM: 99.17%, *etc.*) (Supplementary File 8). Out of which, five algorithms (SVM, RFF, ADB, XGB, and GBM) performed exceptionally well on the cross-validated data (Supplementary File 8). Although there was not much difference in the accuracy of these five ML algorithms, the SVM, RFF, ADB, and XGB performed little bit better over GBM. Further, performance of the TRE algorithm was not better among others for the serotype prediction.

### Independent performance analysis for serotype prediction

**Figure 4:**
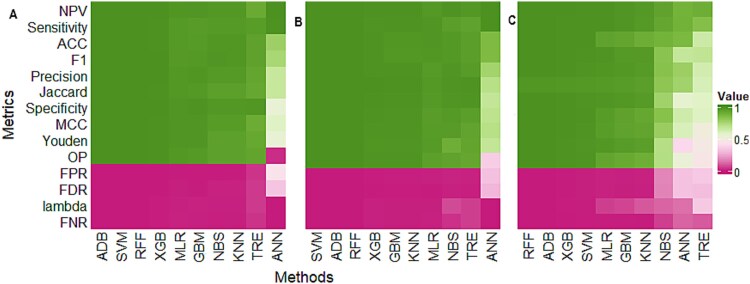
Performance evaluation of the 10 machine learning models for serotype prediction on independent datasets. The results are shown for (A) Afghanistan; (B) India; (C) Zimbabwe datasets. The x-axis represents machine learning methods and y-axis represents performance metrics. For the performance metrics including FPR, FDR, Lambda, and FNR, the lesser value indicates better performance and *vice-versa*, while for other metrics higher value indicates better performance and *vice-versa*.

The ten learning algorithms (trained with 64 *3-mer* features) were further validated on entirely independent datasets those were neither used for model training nor testing. We gathered the VP1 sequence data of FMD virus isolates from the NCBI GenBank database (accessed on 01/11/2023) reported by ten FMD-endemic countries across the world. These countries include Afghanistan, Bangladesh, Bhutan, China, India, Kenya, Nepal, Russia, Turkey, and Zimbabwe and the results are shown in Fig. 4 and Supplementary File 9. A brief description about these datasets is given in Supplementary File 3. Through all the performance metrics including accuracy, sensitivity, precision, *etc.*, the ML algorithms such as SVM, RFF, XGB, and ADB consistently outperformed other techniques across three independent datasets, namely Afghanistan, India, and Zimbabwe (Fig. 4). Though the GBM performed better on cross-validated and test data (Table 2, Supplementary File 9), but its performance was found to be inconsistent over these three independent datasets (Fig. 4). Similar interpretations can be made for other seven independent datasets (Kenya, Nepal, Bangladesh, Russia, Turkey, China, and Bhutan) (Supplementary File 9). Over all the independent datasets, the performance of four ML algorithms (SVM, RFF, XGB, and ADB) was consistently superior in terms of serotype prediction (Supplementary File 9). Further, these best performed algorithms were also assessed based on their ability to predict other components of epidemiology, *i.e.* topotype and lineage.

### Cross-validation performance for topotype prediction

**Figure 5:**
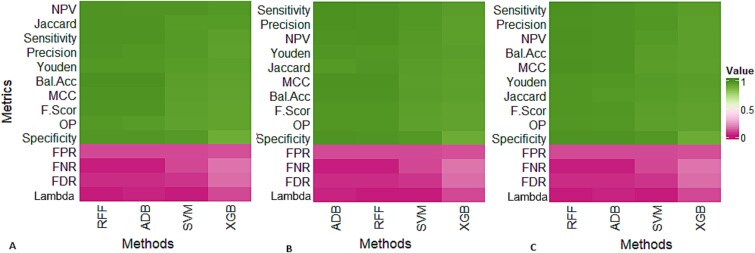
Cross-validated performance evaluation of the SVM, RFF, ADB, and XGB machine learning algorithms for topotype prediction. The results are shown for serotype (A) O; (B) A; (C) Asia 1. The x-axis represents machine learning methods and y-axis represents performance metrics. For the performance metrics including FPR, FDR, Lambda, and FNR, the lesser value indicates better performance and *vice-versa*, while for other metrics higher value indicates better performance and *vice-versa*.

The topotype prediction cross-validated performances of the best four ML approaches are shown in Fig. 5 and Supplementary File 10. There was not much difference in the topotype prediction metrics (accuracy≥96%; precision≥95%; sensitivity and specificity≥94%; F1 and MCC ≥ 95%) of these four learning algorithms (SVM, RFF, XGB, and ADB) for the serotype O (Fig. 5a). However, the RFF and ADB learning algorithms performed somewhat better followed by the SVM and XGB (Fig. 5a). Similar interpretations can be made for topotype predictions of A, Asia 1, and SAT 1–3 serotypes (Fig. 5, Supplementary File 10). Further, slightly different result was observed for serotype C, where the XGB marginally outperformed other algorithms (Fig. 5, Supplementary File 10). Alternatively, the SVM, RFF, XGB, and ADB algorithms performed reasonably better for predicting topotypes of the FMD virus within each serotype.

### Cross-validation performance for lineage prediction

The lineage prediction cross-validated performance of the best four ML algorithms is also carried out for each serotype. Here, lineage prediction results were averaged over the topotypes and presented for each serotype in Table 3. The lineage predictive accuracy of the SVM across all the serotypes was found to be higher (98.22%) except for the C and SAT1–3 serotypes (Table 3). The XGB performed well with higher lineage predictive accuracy for O (94.18%), A (96.18%), Asia 1 (95.67%), and C (99.13%) serotypes compared to others. Not so well performance of the learning algorithms was observed for the SAT serotypes. This might be due to the lack of sufficient lineage training data for these serotypes, as these serotypes are only confined to African regions. Further, the RFF and ADB algorithms performed relatively well (accuracy ≥90%) compared to the SVM and XGB across all the serotypes (Table 3). Overall, the lineage predictive accuracy computed over fivefold-CV for the SVM, RFF, ADB, and XGB was observed to be reasonably satisfactory.

**Table 3:** Repeated 5-fold cross-validation prediction accuracy of SVM, RF, XGB and ADB methods for lineage prediction.

**Methods**	**O**	**A**	**Asia 1**	**C**	**SAT 1**	**SAT 2**	**SAT 3**
**SVM**	98.22 ± 1.49	97.83 ± 1.67	97.50 ± 2.23	90.87 ± 6.15	85.83 ± 7.15	85.19 ± 7.35	84.81 ± 8.15
**RFF**	99.12 ± 0.86	99.17 ± 0.91	99.06 ± 0.91	98.43 ± 1.85	94.17 ± 1.35	95.77 ± 4.85	95.29 ± 3.65
**XGB**	94.18 ± 1.67	96.18 ± 1.37	95.67 ± 1.09	99.13 ± 0.65	89.76 ± 5.35	88.45 ± 4.15	92.6 ± 5.85
**ADB**	99.35 ± 0.77	99.11 ± 0.59	98.87 ± 1.29	95.31 ± 4.15	93.13 ± 2.15	94.38 ± 3.35	94.29 ± 6.15

Values are shown in ($\mu \pm \sigma$) form, where, $\mu$: mean classification accuracy and $\sigma$: standard deviation of accuracy over the data folds; $\mu\ and\ \sigma$ values are averaged over the 100 repetitions.

### MolEpidPred performance on independent data

Four ML algorithms (SVM, RFF, ADB, and XGB) were observed to have superior performance for serotype, topotype, and lineage prediction and thus implemented in our computational tool, MolEpidPred. Next, performance of the MolEpidPred was assessed on five completely independent datasets of FMD virus recently reported in the literature. The predictive accuracies computed through the four algorithms implemented in the MolEpidPred are shown in Table 4. For example, Dahiya *et al.* (2023) reported serotype, topotype, and lineage information of 138 isolates (O serotype) caused the outbreak in India during 2018–2022. The SVM, RFF, XGB, and ADB accurately predicted the serotype and topotype of the 138 virus isolates (accuracy ≥99%) for Dahiya *et al.* data (Table 4). However, the performance of the SVM was found better for lineage prediction followed by the RFF and ADB algorithms.

**Table 4:** Performance of SVM, RFF, XGB and ADB for serotype, topotype, and lineage prediction on recent independent dataset.

**Data**	***n***	**SVM (%)**	**RFF (%)**	**XGB (%)**	**ADB (%)**
Dahiya (2023) **[27]**	138	(100, 100, 100)	(100, 100, 94.20)	(100, 99.27, 84.06)	(100, 100, 94.20)
Eltahir (2024) **[30]**	76	(100, 100, 92.11)	(100, 100, 84.21)	(98.67, 92.10, 81.58)	(100, 100, 80.26)
Jamal (2018) **[29]**	115	(100, 100, 99.13)	(100, 100, 98.26)	(100, 100, 99.13)	(100, 100, 98.26)
Nikiforov (2023) **[26]**	28	(100, 100, 100)	(100, 100, 100)	(100, 96.42, 96.42)	(100, 100, 100)
Zhang (2023) **[38]**	82	(100, 96.34, 93.90)	(100, 96.34, 96.34)	(96.34, 96.34, 95.12)	(100, 96.34, 96.34)

*n*: Number of isolates; (a, b, c): % accuracy for serotype (a), topotype (b), and lineage (c) prediction respectively

For Eltahir *et al.* data, the SVM, RFF, and ADB total correctly predicted the serotype and topotype of the reported virus isolates, while the XGB presented slightly lesser accurate results (Table 4). In terms of lineage prediction, SVM provided better results followed by the RFF for the same dataset. However, the lineage prediction accuracy of all the algorithms was relatively lesser for this dataset compared to other datasets. All the learning algorithms performed exceptionally well in terms of correct prediction of the serotype/topotype/lineage of the virus isolates reported by Jamal *et al.* (Table 4). Further, the SVM, RFF, and ADB algorithms accurately predicted the serotype, topotype, and lineage (accuracy: 100%) of the virus isolates in Nikiforov *et al.* data (Table 4). Here, the XGB accurately predicted serotype of the virus isolates, but its topotype and lineage prediction accuracy was slightly lesser than others. For Zhang *et al.* data, the SVM, RFF, and ADB algorithms provided more accurate results for predicting serotype, topotype, and lineage of the isolates relatively compared to the XGB (Table 4). Overall performance evaluation of the MolEpidPred approach on multiple independent datasets indicated superior performance of the implemented algorithms for prediction of serotype/topotype/lineage of the FMD virus isolates.

### Utility of MolEpidPred for molecular epidemiology of field virus isolates

The raw sequence data of 74 field virus isolates, reported in India during 2018–19, were used in the proposed MolEpidPred approach to predict the serotype/topotype/lineage of the virus causing the outbreaks. The samples were isolated from cloven-hoofed animals across 15 states in India. The prediction results from the MolEpidPred tool are shown in Supplementary File 11. The SVM, RFF, XGB, and ADB algorithms implemented in the MolEpidPred consensually predicted epidemiology of the 60 virus isolates as O/ME-SA/Ind2001 (54) and O/ME-SA/SA2018 (6) (Supplementary File 11). While for 13 isolates, three out of the four learning algorithms predicted the epidemiology as O/ME-SA/Ind2001 (3) and O/ME-SA/SA-2018 (10). For the remaining one isolate (IC6/2019 from Assam), all the algorithms predicted the epidemiology as Asia 1/ASIA/C (Supplementary File 11). Broadly, our approach predicted the epidemiology of the 74 isolates as O/ME-SA/Ind2001 (57), O/ME-SA/SA-2018 (16), and Asia 1/ASIA/C (1). This means FMD virus with Ind2001 and SA-2018 lineages of ME-SA topotype (of serotype O) caused the outbreaks in India during 2018–19.

There is no predictive computational solution available for molecular epidemiology of the FMD virus to benchmark the performance of the MolEpidPred approach on field virus data. Thus, we used phylogenetic analysis implemented in the MEGA (*ver*. 11) to check the results obtained from the MolEpidPred approach. The sequence data of isolates (73) for O serotype were used in the MEGA to find their topotype and lineage, and the results are shown in Supplementary File 11. The findings indicated that topotype and lineage prediction results of the developed approach matched 100% and 96% respectively with that of MEGA results. The Supplementary File 11 contains epidemiological results predicted by the MolEpidPred and validated with the phylogenetic analysis.

### Online prediction server

An online prediction server, *MolEpidPred* (https://nifmd-bbf.icar.gov.in/MolEpidPred), was developed for the researchers across the world for molecular epidemiology of the FMD virus, so that the computational approach could be easily accessed. MolEpidPred is a user-friendly ML interface designed to predict molecular epidemiology of the FMD virus using VP1 sequence data with a single click. The snapshots of server home, algorithm, execution, output, and manual pages of the MolEpidPred web-server are shown in Supplementary File 12. The user interface of the server was designed using HTML, JavaScript, CSS, and Bootstrap. The backend of the web-server was developed using ASP.NET and R-program was integrated as statistical engine for execution of the proposed approach. The intuitive interface of the MolEpidPred enables easy sequence upload *via* copy-paste for fewer isolates in FASTA format or by uploading FASTA file for larger datasets. The user can also select the suitable learning options for the prediction. The predicted epidemiological components of the virus are displayed in tabular format. Since, there was little variation in performance of four models (SVM, RF, ADB, and XGB) in terms of serotype prediction; hence these are implemented in the server. Further, an additional option of simultaneously considering all these models was given, where the result from each technique was provided in WRLFMD recommended notation of ‘Serotype/Topotype/Lineage’. The researchers can use the common results or results from majority of options for highly confident prediction. For offline use of our computational approach, the user can download and utilize the source code and training dataset. Users can download output files in txt/csv format from the predict tab of the server and access interactive visualizations along with comprehensive documentation for effective navigation.

## Discussion

Over the past decade molecular biological techniques (*e.g.* PCR, nucleotide sequencing, *etc.*) have been used in FMD epidemiology [7], which generate high-throughput and quality sequence data. Indeed, conducting molecular epidemiological analyses using these sequence data requires specific skills and in-depth domain knowledge. So, for general use, no web-based computational tool is available in the literature to predict the type, geographical, and genetic groups of the FMD virus. In our previous study, a computational solution was reported only for prediction of serotypes prevalent in Asia [11]. This tool has limited applicability due to narrow serotype coverage, no prediction results on topotype and lineage of the FMD virus, and limited only to Asian region. To address this gap, a novel computational tool called MolEpidPred has been developed that uses state-of-the-art ML techniques and sequence-based *k-mer* features for large-scale molecular epidemiology of the FMD virus. This approach is a three-step process, focusing on predicting the serotype, topotype, and lineage of the virus isolates. Further, the approach is implemented in a web-server, which is an intuitive user interface allowing users to easily upload data and access prediction results in a streamlined manner. This predicted information is crucial for understanding the specific characteristics of the virus responsible for an outbreak and devising appropriate control strategies, including selection of suitable vaccine and containment.

The *k-mer* based sequence features have been successfully implemented in microbiological computational tools [11, 20, 22]. In current investigation, we considered *k-mer* features that were directly generated from the VP1 sequence data due to its simplest execution and easy understanding. However, selection of *k* in *k-mer* features is very crucial, as its higher value increases computational cost multi-fold. So, optimal value of *k* for *k-mer* feature generation was empirically determined as 3 for FMD virus classification using prediction accuracy and runtime as criteria.

Initially, ten learning algorithms were used in serotype prediction to assess their performance on the cross-validated data. All the models except TRE provided better results for serotype prediction when assessed through five-fold CV approach. Precisely, five models (SVM, RF, XGB, GBM, and ADB) performed exceptionally well for serotype prediction with predictive accuracy of ~99%. Here, most of these algorithms implemented boosting technique to reduce the prediction error [23, 43]. Next, the ML algorithms were also assessed on randomly generated test data based on their ability to predict the serotypes of virus isolates. All the algorithms except the TRE model provided reasonably satisfactory results for serotype prediction, when evaluated through 16 performance metrics. Furthermore, the SVM, RFF, XGB, GBM, and ADB outperformed other techniques on random test data across the metrics, similar to the of CV findings.

There might be criticism that the models performed better when assessed on part of the model building data. Therefore, a truly independent validation of the trained models was performed on multiple independent datasets those were neither used for training nor testing. These independent datasets comprised virus isolates reported from 10 FMD-endemic countries, located across the globe. Four ML algorithms (SVM, RFF, XGB, and ADB) consistently outperformed all other models on completely independent datasets. However, the GBM performed better on internal validation and its performance was observed little inconsistent over external independent validation. The superior performance of these four algorithms is also well supported by previous studies including prediction DNA binding proteins [23]. The accurate classification of completely independent isolates served as a clear proof that ML strategy could use high-throughput sequence information to most accurately predict serotypes of isolates never seen before. The above comparative evaluations indicated the outperformance of ML over statistical techniques, which might be due to that former techniques do not require any data distributional assumptions and in fact they learn from the data.

The outperformed models were then considered for further prediction of topotype and lineage of the virus isolates in subsequent steps. The rationale was that success of topotype and lineage prediction depends on serotype prediction performance of the models. Based on the serotype prediction results, four ML algorithms (SVM, RFF, XGB, and ADB) were chosen for second and third steps of topotype and lineage prediction, respectively. The topotype and lineage prediction of these chosen algorithms were found satisfactory when evaluated on cross-validated and random test data. Therefore, all these four ML algorithms were implemented in our MolEpidPred computational tool.

The effectiveness of the proposed MolEpidPred tool was assessed on recently reported FMD virus isolates in Dahiya *et al.* [27], Eltahir *et al.* [30], Jamal *et al.* [29], Nikiforov *et al.* [26], and Zhang *et al.* [38] studies. The sequence data of these isolates were retrieved from the GenBank database and serotype, topotype, and lineage information were obtained from their publications. The predictive performance of our approach was compared with the results reported in the respective literature. The findings suggested that all the four ML algorithms implemented in our developed tool most accurately predicted the components of epidemiology (*i.e.* serotype/topotype/lineage). The completely independent validation of the MolEpidPred approach indicated its effective use in molecular epidemiology of FMD virus.

We also demonstrated the utility of our developed MolEpidPred tool in field setup through applying on novel virus isolates reported from various outbreaks in India during 2018–19. The virus isolates were isolated from the foot and epithelium samples of infected cattle, buffalo, goat, and pig and sequenced at our ICFMD laboratory facility. The MolEpidPred tool predicted the epidemiology of the 74 virus isolates as O/ME-SA/Ind2001 (57), O/ME-SA/SA-2018 (16) and Asia 1/ASIA/C (1). This finding is supported by the evidence that FMD outbreak India in 2018–19 caused by the Ind2001 and SA-2018 lineages of ME-SA topotype of the O serotype [28, 46]. The Ind2001 FMD virus lineage was first reported in India during 2001 [47] and then it escaped to other parts of world during 2013–2022 that have led to outbreaks in North Africa [48], Middle East [49], Southeast Asia [50], and Russia and Kazakhstan [26] due to the transboundary movement of animals. The comparison of our findings with phylogenetic analysis indicated that accurate matching of topotype and lineage prediction of the MolEpidPred with the latter. Unlike phylogenetic analysis, the MolEpidPred tool is easy to use, provides quick prediction results, and only needs sequence data of query isolates. This demonstrated the effective use of the developed approach in field setup for molecular epidemiology of the FMD virus.

For the better use of the developed computational approach among the FMD researchers and policy makers across the globe, we developed one freely accessible web-based prediction server. This tool can handle the raw sequence data, which sometimes contain non-nucleotide or missing bases. Further, the developed web-application is first of its kind and provides quick computational solution to the FMD researchers across the globe without delving deeper into computational complexities. However, it is important to recognize that challenges such as the need for high-quality input data, potential algorithm biases, and the evolving nature of FMD virus strains could impact the overall performance and applicability of the MolEpidPred platform in diverse contexts.

## Conclusion

FMD is an economically devastating disease affecting the livestock health and productivity. Instant and quick molecular epidemiological prediction of the FMD virus would help in tracking the virus infection and evolution. Therefore, this study presents a novel computational strategy and innovative tool for molecular epidemiology of the FMD virus, which intends to speed up and improve the FMD management and preventive initiatives. Besides, this study proved the concept of using high-throughput sequencing coupled with ML for deeper understanding into the complex epidemiology of the virus. The findings indicated that the SVM, RFF, XGB, and ADB were better for predicting the epidemiological components of the FMD virus. Further, these techniques were implemented in the developed MolEpidPred web-prediction server. The comparative evaluation on test and independent datasets as well as validation on field virus isolates data suggested the superior performance of MolEpidPred on secondary and primary datasets. To the best of our knowledge, this is the first ever computational tool designed for molecular epidemiology of the FMD virus. As global livestock markets continue to expand, the utility of this tool will become increasingly important. Thus, the proposed tool has the potential to be used across the FMD-endemic countries *via* online-prediction server (https://nifmd-bbf.icar.gov.in/MolEpidPred) to predict molecular epidemiology of the virus. Stakeholders including veterinarians, researchers, and policymakers are encouraged to adopt this platform for epidemiology of the FMD virus.

The major limitations of the developed computational platform are its dependency on high-quality input data and potential algorithm biases. The future challenge is to predict the sub-lineages of the virus isolates, which could not be implemented in the current platform due to high-computational time requirement. Furthermore, on-going research and refinement will ensure the platform’s accuracy and adaptability to address future challenges in global livestock health. Additional and novel pathogens of infectious diseases (*e.g.* COVID19, lumpy skin disease, *etc.*) can be incorporated into the tool through appropriate model training, offering the potential of this technology to rapidly use in future infectious diseases in humans and animals.

**Key Points**

- Reported a novel and innovative computational tool, MolEpidPred, for large scale molecular epidemiology of most devastating animal disease pathogen, FMD virus.
- MolEpidPred, a three-stage approach, achieved reasonable accuracy for serotype, topotype, and lineage prediction of the FMD virus.
- Independent validation followed by testing on wet-lab data confirmed the reliability, generalized predictive ability, and utility of the MolEpidPred tool.
- Computational tool is implemented in an online prediction server, freely accessible at https://nifmd-bbf.icar.gov.in/MolEpidPred for general use.
- MolEpidPred web-prediction server provides a quick and easy way for large-scale molecular epidemiology of the FMD virus isolates across the globe.

## Supplementary Material

Supplementary_Materials_elaf001

## Data Availability

The secondary and primary datasets used in this study are available in https://github.com/sam-dfmd/MolEpidPred_Data. The source code and training data of the web server is available at https://nifmd-bbf.icar.gov.in/MolEpidPred.
