# Supplementary material for: MolEpidPred: a novel computational tool for the molecular epidemiology of foot-and-mouth disease virus using VP1 nucleotide sequence data
Source: Brief Funct Genomics. 2025 Mar 5;24:elaf001. doi: 10.1093/bfgp/elaf001 (PMC11881699; doi:10.1093/bfgp/elaf001)

**Supplementary Files**

of

MolEpidPred: A Novel Computational Tool for the Molecular Epidemiology of Foot-and-Mouth Disease Virus using VP1 Nucleotide Sequence Data

Samarendra Das^1*^, Utkal Nayak^1^, Soumen Pal^2^, Saravanan Subramaniam^1^

^1^ICAR-National Institute on Foot and Mouth Disease, International Centre for Foot and Mouth Disease, Arugul, Bhubaneswar-752050, India

^2^Division of Computer Application, ICAR-Indian Agricultural Statistics Research Institute, New Delhi-110012, India

^*^Corresponding author: [Samarendra.Das@icar.gov.in](mailto:Samarendra.Das@icar.gov.in)

**Contents**

| **Sl. No.** | **Supplementary file** | **Topics** | **Page No.** |
| --- | --- | --- | --- |
| 01 | SupplementaryFile1 | Molecular epidemiology of the FMD virus | 2 |
| 02 | SupplementaryFile2 | Sequence data collection | 3 |
| 03 | SupplementaryFile3 | Data from independent countries | 4 |
| 04 | SupplementaryFile4 | Sample collection and data generation for field isolates | 5 |
| 05 | SupplementaryFile5 | Determination of optimal ‘k’ in *k-mer* | 6 |
| 06 | SupplementaryFile6 | List of optimal 3-mer features | 7 |
| 07 | SupplementaryFile7 | Machine learning techniques | 8-14 |
| 08 | SupplementaryFile8 | Cross-validated performance analysis of serotype prediction models | 15 |
| 10 | SupplementaryFile9 | Independent evaluation of trained serotype prediction machine learning models | 16 |
| 11 | SupplementaryFile10 | Cross-validation performance analysis of trained topotype prediction machine learning models | 17 |
| 12 | SupplementaryFile11 | MolEpidPred web-prediction server for epidemiology of field FMD virus isolates | 18-20 |
| 13 | SupplementaryFile12 | MolEpidPred web-prediction server | 21-23 |

**SupplementaryFile1. Components of molecular epidemiology of Foot-and-mouth Disease (FMD) virus**

Molecular epidemiology is a branch of epidemiology emerged out of the usage of recent molecular biological techniques into epidemiological studies of the infectious diseases. Here, molecular epidemiology of the FMD identifies infectious disease causing virus variety, its source, reservoirs, ancestors, circulation pattern, transmission pattern, transmission probability, and transmission order, *etc*. Thus, knowing in-depth insight into these factors is necessary for developing more effective prevention and FMD control strategies and policies. For instance, knowing the proper serotype of the FMD virus will help to choose the proper vaccine candidate. In this context, we tried to present the molecular epidemiology of the FMD virus in the following figure.


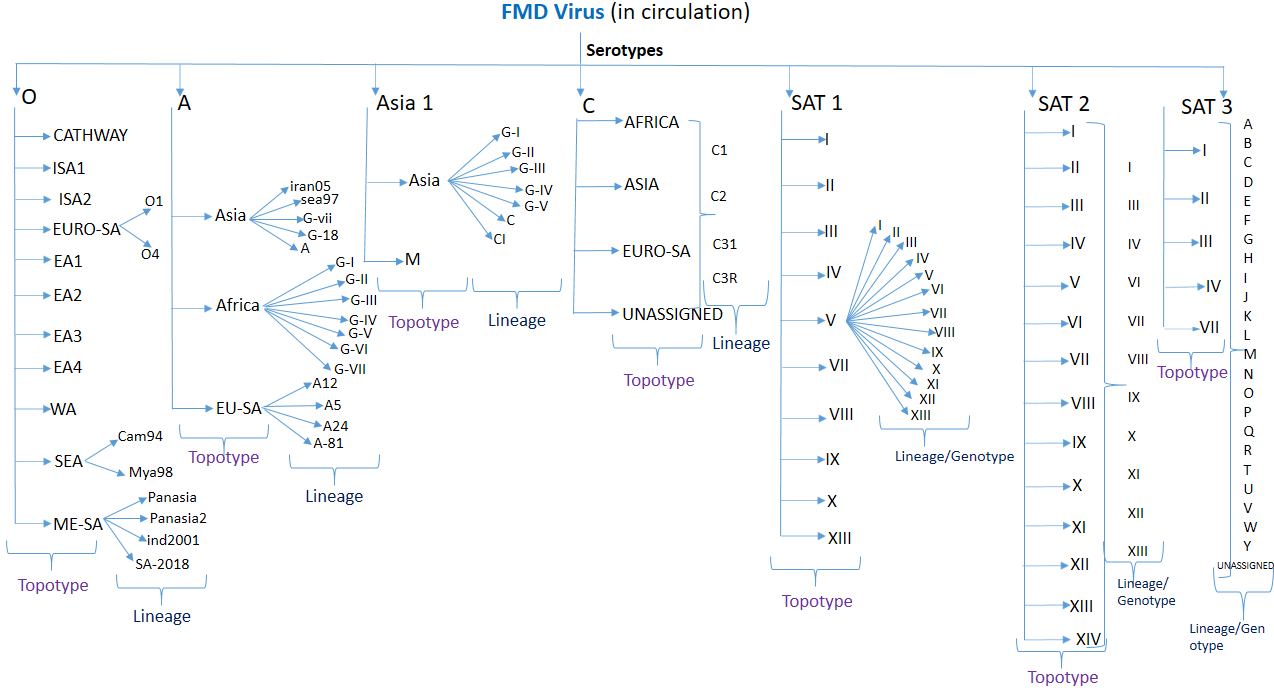


**Figure S1.** Pictorial representation of molecular epidemiology of the FMD virus.

The FMD virus is in global circulation with seven different varieties, known as serotypes (i.e., O, A, Asia 1, C, SAT 1, SAT 2, and SAT3). Among all the serotypes, the serotype O causes maximum infection in the world. For instance, ~92% of total FMD outbreak in India is caused by this serotype. Further, each serotype is divided into several topotypes based on their geographical distributions. For example, serotype O is divided into 11 topotypes, i.e., CATHWAY, ISA1, ISA2, Euro-SA, EA1, EA2, EA3, EA4, WA, SEA, and ME-SA. The FMD virus of serotype O with ME-SA and SEA topotypes mostly dominate the outbreak scenarios in Asia. Then, the virus isolates within each topotype is further divided into multiple lineages based on their sequence similarity (>85% for O, A, C, and Asia 1) or (>80% for SAT 1, SAT 2, and SAT 3) in their VP1-coding region. For example, the ME-SA topotype FMD virus isolates is divided into four lineage groups including PANASIA, PANASIA2, Ind2001, and SA-2018.

**SupplementaryFile2. Sequence retrieval from the public domain databases.**

**Table S1. Total FMD virus isolates sequence (VP1) data retrieved from public domain databases.**

| **Sl. No.** | **Serotype** | **Serotype (sample size)** | **Topotype**  **(sample size)** | **Lineage**  **(sample size)** |
| --- | --- | --- | --- | --- |
| 01 | O | 4209 | 350 | 327 |
| 02 | A | 1853 | 52 | 57 |
| 03 | ASIA1 | 613 | 76 | 54 |
| 04 | C | 153 | 144 | 76 |
| 05 | SAT1 | 416 | 45 | 19 |
| 06 | SAT2 | 623 | 65 | 19 |
| 07 | SAT3 | 94 | 51 | 52 |
| **Total** | | **7961** | **783** | **604** |

**SupplementaryFile3. Data from Independent countries.**

**Table S1. Independent datasets, reported from FMD-endemic countries, used for validation of trained models.**

| **SN.** | **Data** | **#Virus isolates** | **Description** | **Accession ID** |
| --- | --- | --- | --- | --- |
| 1 | Afghanistan | 401 (A: 128; Asia 1: 25; O: 248) | VP1 sequence of FMDV isolates from Afghanistan | HQ439230-38, DQ165035,…, HQ439291 |
| 2 | Bangladesh | 274 (A: 53; Asia 1: 39; C: 2; O: 180) | VP1 sequence of FMDV isolates from Bangladesh | KT960948, KR869774, …, N447113 |
| 3 | Bhutan | 52 (A: 6; Asia 1: 3; O: 40; C: 3) | VP1 sequence of FMDV isolates from Bhutan | MT276848, MT276847, EU414525, …, MK390943 |
| 4 | China | 77 (A: 6; Asia 1: 7; O: 64) | VP1 sequence of FMDV isolates from China | MT447399, MH791315-18, …, AJ131663 |
| 5 | India | 345 (A: 85; Asia 1: 110; O: 150) | VP1 sequence of FMDV isolates from India | KY780958, HQ127678, …, MT909720 |
| 6 | Kenya | 373 (SAT1: 147; SAT2: 155; C: 2; O: 49; A: 20) | VP1 sequence of FMDV isolates from Kenya | MH882578-611, KP263446, …, MH882567 |
| 7 | Nepal | 120 (A: 1; Asia 1: 1; C: 5; O: 120) | VP1 sequence of FMDV isolates from Nepal | QOW40785, QOW40784, AQZ26542, …, MK390954 |
| 8 | Russia | 44 (O: 33; A: 6; Asia 1: 5) | VP1 sequence of FMDV isolates from Russia | AJ318850, MG972585, …, DQ121402 |
| 9 | Turkey | 517 (O: 165; A: 318; Asia 1: 34) | VP1 sequence of FMDV isolates from Turkey | AJ296325, KY091287, …, EU553915 |
| 10 | Zimbabwe | 260 (SAT1: 114; SAT2: 86; SAT3: 60) | VP1 sequence of FMDV isolates from Zimbabwe | MT385618, MT385617, …, MT219952 |

The above curated secondary datasets are available at <https://github.com/sam-dfmd/MolEpidPred_Data> for reproducing the results.

**SupplementaryFile4. Sample collection and data generation for 74 field isolates**

We also demonstrated the utility of the developed computational solution in field-setup through generating primary sequence (VP1 region) data at the ICFMD laboratory, a Food and Agriculture Organization (FAO) reference laboratory for the FMD located in India. For this purpose, clinical samples from various FMD-outbreaks areas of India, during the year 2018-19, were considered. Here, tongue and foot epithelium samples from cloven-hoofed animals including cattle, buffalo, goat, and pig, were collected by the state FMD research centres and Veterinary departments, following the World organization of animal health approved guidelines across various locations in India covering Punjab, Madhya Pradesh, Maharashtra, Karnataka, Tamil Nadu, Assam, Tripura, West Bengal, Puducherry, Chhattisgarh, and Odisha.

First, the epithelial tissue samples were ground with sterile mortar and pestle to prepare a 10% suspension in phosphate buffer saline. Then, total RNA was extracted from the suspensions prepared from the FMD suspected clinical samples (tongue or foot epithelium) using the Viral RNA Mini Kit (Qiagen, Germany) as per the manufacturer’s instructions. The extracted RNA was used for the synthesis of cDNA using an oligod(T)_15_ primer and Moloney murine leukemia virus reverse transcriptase enzyme (Promega, USA). The VP1 structural protein coding region was amplified using a pair of primer (L463-NK61) and *pfu*DNA polymerase enzyme. The thermal conditions followed were 95^o^C for 2 min, 35 cycles of denaturation at 95^o^C for 30 sec, annealing at 53^o^C for 30 sec and extension at 72^o^C for 5 min followed by final extension step of 72^o^C for 10 min. The PCR product was purified using QIAquick gel extraction kit (Qiagen, Germany) and sequences were resolved using primers ARS4 and NK61 on ABI-3500xL DNA analyzer (Applied Biosystem, USA) using the Bigdye V3.1 terminator kit. The nucleotide sequence data of the 74 FMD Virus isolates are supplied at <https://github.com/sam-dfmd/MolEpidPred_Data/blob/main/Wetlab_SequenceData.txt>.

**SupplementaryFile5. Determination of optimal ‘*k*’ in *k-mer* feature generation approach**

In machine learning, the sequences cannot be directly used for model building, therefore, features will be generated and subsequently used. For this purpose, k-mer technique will be used and described as follows.

The k-mer technique generates features from the VP1 nucleotide sequence data of the FMD virus isolates. *k-mers* are 'k'-length substrings found in biological sequences. It is possible to utilise a collection of *k-mers*' frequency as a "signature" of the underlying sequence in a genomic region (*e.g*., VP1 region). Additionally, it can be utilised as a preliminary analysis before an alignment. A sequence of length *L* typically has (*L-k+1) k*-mers and 𝑛𝑘 potential *k-mers*. (where, n = number of possible monomers, *e.g*., 4 for DNA bases, *i.e*., A, T, G, and C). The process of feature generation is briefly described in Figure C.


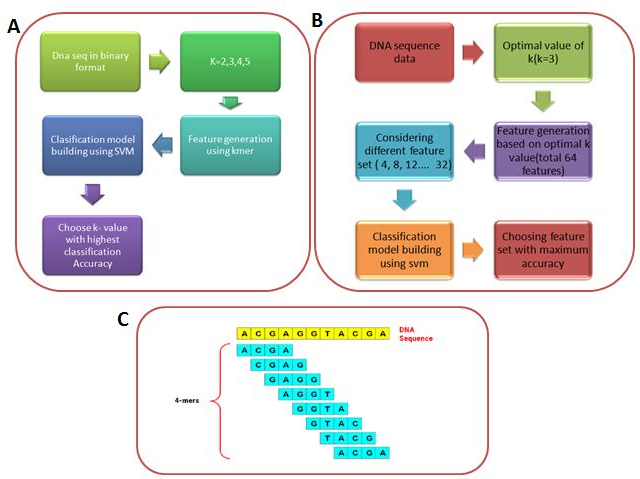


**Figure S1.** Feature generation using k-mer technique. (A) Empirical approach for optimal K determination. (B) Empirical approach for optimal feature set selection. (C) Feature generation.

Since there is no set method or guideline for choosing the best k values, therefore, we developed an empirical approach for optimal *K* value determination. First, a set of *K* values, K = 1, 2, 3, 4, …,16 was taken and the DNA sequences were initially recorded in binary format for this range of *K* values. Then, using the *k*-*mer* technique, features were generated for each value of *K*. For example, for k=2, the number of features will be 16 (=4^2^). Then, the features counts data for individual virus sequences were obtained, as shown in Figure C. Then, a classification model, *e.g*., Support Vector Machine (SVM) was trained on the feature count data generated for that K value to compute the classification accuracy and required runtime (*i.e*., computational time required to execute the computer program). This process was repeated for all the values, K=1, 2, 3,…, 16 and subsequently classification accuracies were obtained. Finally, that *K* value was selected as optimal value for feature generation that provided the highest level of classification accuracy. The steps of the proposed algorithm are shown in Figure A. The accuracies *vs*. k-values were plotted and to visualize the optimal value of the *k.*

**SupplementaryFile6. Optimal 3-mer feature set.**

| **Sl. No.** | **Feature** | **Sl. No** | **Feature** | **Sl. No.** | **Feature** | **Sl. No.** | **Feature** |
| --- | --- | --- | --- | --- | --- | --- | --- |
| 01 | AAA | 17 | CAA | 33 | GAA | 49 | TAA |
| 02 | AAC | 18 | CAC | 34 | GAC | 50 | TAC |
| 03 | AAG | 19 | CAG | 35 | GAG | 51 | TAG |
| 04 | AAT | 20 | CAT | 36 | GAT | 52 | TAT |
| 05 | ACA | 21 | CCA | 37 | GCA | 53 | TCA |
| 06 | ACC | 22 | CCC | 38 | GCC | 54 | TCC |
| 07 | ACG | 23 | CCG | 39 | GCG | 55 | TCG |
| 08 | ACT | 24 | CCT | 40 | GCT | 56 | TCT |
| 09 | AGA | 25 | CGA | 41 | GGA | 57 | TGA |
| 10 | AGC | 26 | CGC | 42 | GGC | 58 | TGC |
| 11 | AGG | 27 | CGG | 43 | GGG | 59 | TGG |
| 12 | AGT | 28 | CGT | 44 | GGT | 60 | TGT |
| 13 | ATA | 29 | CTA | 45 | GTA | 61 | TTA |
| 14 | ATC | 30 | CTC | 46 | GTC | 62 | TTC |
| 15 | ATG | 31 | CTG | 47 | GTG | 63 | TTG |
| 16 | ATT | 32 | CTT | 48 | GTT | 64 | TTT |

**SupplementaryFile7. Machine learning techniques used in this study.**

1. ***Support Vector Machine***

The Support Vector Machine (SVM) supervised learning technique (Cortes and Vapnik, 1995) has been extensively used in the area of computational biology and bioinformatics (Chou and Cai, 2002; Das et al., 2017; Das and Rai, 2020). In the context of virus classification, SVM has already been used in earlier studies (Griffel et al., 2018; Öz and ÖE, 2019). The kernel functions play vital role as far as the predictive ability of SVM is concerned. For instance, the linear kernel function, the input dataset is transformed into a high-dimensional feature space in which the observations of different classes are linearly separable by optimal separating hyper plane. We also employed SVM for prediction purpose in this study. Here, 70% of the model building data was used for model training and remaining 30% was used for model testing. This random splitting of data was repeated for 500 times. Besides, we also used five-fold cross validation technique to compute the predictive accuracy. To implement this classifier, *svm* function available in *e1071* package (Meyer et al., 2022) of R-software (R Core Team, 2022) was executed.

1. **Random Forest**

Random Forest **(**RFF) is an ensemble learning method, collection of multiple classification trees [52], and each tree is constructed on data sample drawn from a training set with replacement, called the bootstrap sample. As each tree is built on bootstrap sample, ~1/3^rd^ of observations do not play any role in the construction of tree and are called as Out-Of-Bag (OOB) instances [53]. The OOB samples are mainly used for measuring the prediction error. For prediction purpose, each constructed tree of the RF votes each test sample to one of the predefined multi-classes and further class of the test sample is predicted by label of majority of votes [53]. RF algorithms have two main hyper-parameters, *i.e*., *mtry* (number of variables to choose at each node for splitting) and *ntree* (number of trees to construct in the forest), which need to be tuned before training to achieve maximum prediction accuracy. For tuning the RF, we executed *tune.rf* function implemented in *randomForest* R-package [54]. Here, various combinations of *ntree* and *mtry* parameters were tested and the combination which provided lowest OOB-error rate was considered as the optimal value and used for model training. For training model, the *randomForest* function  available in the *randomForest* R package [54] was executed with the tuned parameters.

1. **AdaBoost**

The AdaBoost (ADB) is a powerful ensemble learning technique extensively used for classification tasks. The fundamental idea behind the ADB is to combine multiple weak learners (*e.g*., decision trees) with on different weighted iterations of the dataset to build a robust and accurate model. It works iteratively by assigning higher weights to misclassified instances in each round, thus focusing on the difficult-to-classify instances. Subsequent weak learners are then trained to prioritize these instances, gradually improving the overall model performance. By iteratively adjusting the weights of misclassified samples, the ADB effectively learns from its mistakes and builds a strong classifier that can generalize well to unseen data. The ADB is widely known for its capacity to handle complicated datasets and enhance the performance of weak learners by merging them into a strong learner, resulting in the creation of a reliable and accurate classification model. To implement the ADB model, we executed the *booster* function implemented in the *rbooster* R package (*ver*. 1.1.0).

1. **XGBoost**

The XGBoost (XGB) is a sophisticated machine learning algorithm that is well-known for its effectiveness and efficiency while processing structured data. It is especially effective for classification problems and is a member of the gradient boosting algorithm family. Decision trees are progressively added to the ensemble by the XGB, with each new tree fixing the mistakes caused by the preceding ones. In an effort to consistently raise the predictive performance of the model, it minimises a loss function using the gradient descent optimisation technique. Regularisation strategies are also included in XGBoost to avoid over-fitting and it offers a range of hyper-parameters for fine-tuning, which further enhances its adaptability to diverse datasets. To execute the XGB learning technique, the *xgb.train* R function of the xgboost R package (*ver*. 1.7.6.1) was implemented.

1. **Decision Tree**

Decision Tree (TRE) is a supervised machine learning technique mostly used for solving classification problems. It is a tree-structured classifier, where internal nodes represent the features of a dataset, branches represent the decision rules and each leaf node represents the outcome/class. In this model, there are two nodes, which are the Decision Node and Leaf Node. Decision nodes are used to make any decision and have multiple branches, whereas Leaf nodes are the output of those decisions and do not contain any further branches. The decisions or the test are performed on the basis of features of the given dataset. Recently, it was used in Veterinary science for pathogen identification (Lowie et al., 2021). Here, 70% of the model building data was used for model training and remaining 30% was used for model testing. This random splitting of data was repeated for 500 times to compute various model performance criteria. Besides, we also used five-fold cross validation technique to compute the predictive accuracy. To implement this classifier, *rpart* function available in *rpart* package (Therneau and Atkinson, 2022) of R-software(R Core Team, 2022) was executed.

1. **Gradient Boosting Machine**

Gradient Boosting Machine (GBM) is a powerful machine learning technique that builds predictive models by combining the strengths of multiple decision trees. Unlike traditional decision tree algorithms, GBM sequentially trains a series of weak learners (*i.e.*, shallow decision trees) with each subsequent tree aiming to correct the errors made by its predecessors. This iterative process involves minimizing a predefined loss function by adjusting the parameters of the weak learners, such as the tree structure and the weights assigned to different data points. By continuously focusing on the instances that are difficult to predict, the GBM gradually constructs a robust ensemble model capable of capturing complex relationships within the data. To execute the GBM learning technique, the *gbm* R function of the gbm R package (*ver*. 2.1.9) was implemented.

1. **K- Nearest Neighbour**

K-Nearest Neighbour (KNN) is one of the simplest supervised machine learning algorithms extensively used for classification. K-NN is a non-parametric method, i.e., does not make any explicit assumptions about the distribution of underlying data. Further, it only assumes the similarity between the new samples and available samples (in training data) and put the new samples into the category/class that is most similar to the available categories. Alternatively, K-NN algorithm stores all the available data and classifies a new data point based on the similarity. Recently, KNN has been adopted in virus detection and classification (Zhang et al., 2019; Arslan et al., 2021). The major steps in KNN technique:

Step-1: Select the number K of the neighbors

Step-2: Calculate the Euclidean distance of K number of neighbors

Step-3: Take the K nearest neighbors as per the calculated Euclidean distance.

Step-4: Among these k neighbors, count the number of the data points in each category.

Step-5: Assign the new data points to that category for which the number of the neighbor is maximum.

To build the KNN model FMD virus classification, the data was randomly splitted into 70% and 30% for training and testing respectively. This procedure was repeated for 500 times to compute various model performance criteria. Besides, we also used five-fold cross validation technique to compute the predictive accuracy. To implement this classifier, *knn* function available in *class* package (Venables and Ripley, 2002) of R-software (R Core Team, 2022) was executed.

1. **Artificial Neural Network**

Neural networks machine learnings are loosely representative of the human brain learning. An Artificial Neural Network (ANN) consists of Neurons which in turn are responsible for creating layers. These Neurons are also known as tuned parameters. The output from each layer is passed on to the next layer. There are different nonlinear activation functions to each layer, which helps in the learning process and the output of each layer. The output layer is also known as terminal neurons.A neuron in an ANN is: set of input values and associated weights; function that sums the weights and maps the results to an output. Recent studies showed that ANN is potential machine learning option for virus detection and classification using sequence data (Brion et al., 2005; Dasari et al., 2022). In this study, ANN was trained and tested on 70% and 30% of data respectively obtained through random splitting. This procedure was repeated for 500 times to compute various model performance criteria. Besides, we also used five-fold cross validation technique to compute the predictive accuracy. To implement this classifier, *neuralnet* function available in *neuralnet* package (Fritsch et al., 2019) of R-software (R Core Team, 2022) was executed.

1. ***Multi-class Logistic Regression***

Multinomial Logistic Regression (MLR) is natural extension of logistic regression to multi-class classification problems. Logistic regression, by default, is limited to two-class classification problems. The MLS algorithm is an extension to the logistic regression model that involves changing the loss function to cross-entropy loss and predict probability distribution to a multinomial probability distribution to natively support multi-class classification problems. It has been used in virus classification, as evident from the literature (Black et al., 2007). To build MLS model, it was trained and tested on 70% and 30% of data respectively obtained through random splitting. This procedure was repeated for 500 times to compute various model performance criteria. Besides, we also used five-fold cross validation technique to compute the predictive accuracy. To implement this classifier, *multinom* function available in *nnet* package (Fritsch et al., 2019) of R-software (R Core Team, 2022) was executed.

1. **Naïve Bayes**

The Naive Bayes (NBS) is a supervised classification technique for binary and multiclass classification problems. It is used to classify upcoming samples by assigning class labels to instances/records using conditional probability. It is called NB or idiot Bayes because the calculations of the probabilities for each class are simplified to make their calculations tractable.Rather than attempting to calculate the probabilities of each attribute value, they are assumed to be conditionally independent given the class value.In this study, NB model was trained and tested on 70% and 30% of data respectively obtained through random splitting. This procedure was repeated for 500 times to compute various model performance criteria. Besides, we also used five-fold cross validation technique to compute the predictive accuracy. To implement this classifier, *naiveBayes* function available in *e1071* package (Meyer et al., 2022) of R-software (R Core Team, 2022) was executed.

The above listed machine learning techniques used in this study (for FMD virus molecular epidemiology) are summarized in following table.

**Table S1**. List of the machine learning techniques used in this study.

| **Sl. No.** | **Machine learning technique** | **R package** | **Version** | **R functions** | **Reference** |
| --- | --- | --- | --- | --- | --- |
| **1** | Support Vector Machine (SVM) | e1071 | 1.7-14 | svm | Meyer et al., 2022 |
| **2** | Random Forest (RFF) | randomForest | 4.7-1.1 | randomForest | Liaw and Wiener (2002) |
| **3** | *K*-nearest neighbour (KNN) | class | 7.3-22 | knn | Venables & Ripley, 2002 |
| **4** | Artificial Neural Network (ANN) | neuralnet | 1.44.2 | neuralnet | Fritsch et al., 2019 |
| **5** | Decision Tree (Tree) | rpart | 4.1.19 | rpart | Therneau and Atkinson, 2022 |
| **6** | Multi-Class Logistic Regression (MLR) | nnet | 7.3-19 | multinom | Venables & Ripley, 2002 |
| **7** | Gradient Boosting Machine (GBM) | gbm | 2.1.9 | gbm | Ridgeway, 2024 |
| **8** | Naïve Bayes (NB) | e1071 | 1.7-14 | naiveBayes | Meyer et al., 2022 |
| **9** | AdaBoost (ADB) | rbooster | 1.1.0 | booster | Saglam, 2021 |
| **10** | XGBoost (XGB) | xgb.train | 1.7.6.1 | xgboost | Chen et al. (2023) |

For the better performance of the machine learning models, it is necessary to tune the model parameters. Here, we tuned the model parameters w.r.t to the model building data and computed optimal model parameters are given in Table S2. Further, these optimal parameters were used in machine learning models training.

**Table S2.** Parameters settings for machine learning techniques.

| **ML Techniques** | **R packages** | **Parameters** |
| --- | --- | --- |
| SVM | e1071 | kernel = "linear", gamma = 0.5, cost = 1, tolerance = 0.001, epsilon = 0.1 |
| RFF | randomForest | ntry = 4, mtree=500 |
| KNN | class | K = 4 |
| ANN | neuralnet | hidden = 3 |
| TRE | rpart | Default parameter setting |
| MLS | nnet | Default parameter setting |
| NBS | e1071 | Default parameter setting |
| XGB | xgb.train | max_depth=5, eta=0.3, gamma=1, colsample_bytree=0.6, min_child_weight=3, subsample=0.6, objective="multi:softmax", num_class=7 |
| ADB | booster | max_iter = 100, lambda = 1, bag_frac = 0.5, p_weak = NULL, weighted_bootstrap = TRUE |
| GBM | gbm | n.trees = 100, interaction.depth = 3, shrinkage = 0.1 |

**References**

Cortes, C. and Vapnik, V. (1995) Support-Vector Networks. Machine Learning, 20, 273-297.doi.org/10.1007/BF00994018

Chou, K.C. and Cai, Y.D. (2002) Using Functional Domain Composition and Support Vector Machines for Prediction of Protein Subcellular Location. The Journal of Biological Chemistry, 277, 45765-45769. doi.org/10.1074/jbc.M204161200

Das S, Meher PK, Rai A, Bhar LM, Mandal BN. Statistical Approaches for Gene Selection, Hub Gene Identification and Module Interaction in Gene Co-Expression Network Analysis: An Application to Aluminum Stress in Soybean (Glycine max L.). PLoS One. 2017 Jan 5;12(1):e0169605. doi: 10.1371/journal.pone.0169605.

Das S, Rai SN. Statistical Approach for Biologically Relevant Gene Selection from High-Throughput Gene Expression Data. Entropy (Basel). 2020 Oct 25;22(11):1205. doi: 10.3390/e22111205.

Öz E, Aşkın ÖE. Classification of Hepatitis Viruses from Sequencing Chromatograms Using Multiscale Permutation Entropy and Support Vector Machines. Entropy. 2019; 21(12):1149. <https://doi.org/10.3390/e21121149>

Griffel, L.M., Delparte, D., Edwards, J. (2018). Using Support Vector Machines classification to differentiate spectral signatures of potato plants infected with Potato Virus Y, Computers and Electronics in Agriculture, 153, 318-324. doi.org/10.1016/j.compag.2018.08.027.

Meyer D., Dimitriadou E., Hornik K., Leisch F., Weingessel A. (2022).  e1071: Misc Functions of the Department of Statistics, Probability Theory Group (Formerly: E1071), TU Wien_. R package version 1.7-11. <https://CRAN.R-project.org/package=e1071>.

R Core Team (2022). R: A language and environment for statistical computing. R Foundation for Statistical Computing, Vienna, Austria. <https://www.R-project.org>

Lowie T, Callens J, Maris J, Ribbens S, Pardon B. Decision tree analysis for pathogen identification based on circumstantial factors in outbreaks of bovine respiratory disease in calves. Prev Vet Med. 2021 Nov;196:105469. doi: 10.1016/j.prevetmed.2021

Therneau T, Atkinson B (2022). rpart: Recursive Partitioning and Regression Trees. R package version 4.1.16, <https://CRAN.R-project.org/package=rpart>

Zhang Z, Cai Z, Tan Z, Lu C, Jiang T, Zhang G, Peng Y. Rapid identification of human-infecting viruses. TransboundEmerg Dis. 2019 Nov;66(6):2517-2522. doi: 10.1111/tbed.13314. Epub 2019 Aug 12.

Arslan H, Arslan H. A new COVID-19 detection method from human genome sequences using CpG island features and KNN classifier. Engineering Science and Technology, an International Journal. 2021 Aug;24(4):839–47. doi: 10.1016/j.jestch.2020.12.026. Epub 2021

Venables, W. N. & Ripley, B. D. (2002) Modern Applied Statistics with S. Fourth Edition. Springer, New York. ISBN 0-387-95457-0

Brion G, Viswanathan C, Neelakantan TR, Lingireddy S, Girones R, Lees D, Allard A, Vantarakis A. Artificial neural network prediction of viruses in shellfish. Appl Environ Microbiol. 2005 Sep;71(9):5244-53. doi: 10.1128/AEM.71.9.5244-5253.2005.

Dasari CM, Bhukya R. Explainable deep neural networks for novel viral genome prediction. ApplIntell (Dordr). 2022;52(3):3002-3017. doi: 10.1007/s10489-021-02572-3. Epub 2021 Jun 25. PMID: 34764607; PMCID: PMC8232563.

Fritsch S, Guenther F, Wright M (2019). neuralnet: Training of Neural Networks. R package version 1.44.2, <https://CRAN.R-project.org/package=neuralnet>

Black LE, Brion GM, Freitas SJ. Multivariate logistic regression for predicting total culturable virus presence at the intake of a potable-water treatment plant: novel application of the atypical coliform/total coliform ratio. Appl Environ Microbiol. 2007 Jun;73(12):3965-74. doi: 10.1128/AEM.02780-06.

Fan J and Fan Y (2008). High-dimensional classification using features annealed independence rules. Ann. Statist 36, 2605–2637.

A. Liaw and M. Wiener (2002). Classification and Regression by randomForest. R News

2(3), 18--22.

Chen T, He T, Benesty M, Khotilovich V, Tang Y, Cho H, Chen K, Mitchell R, Cano I,

Zhou T, Li M, Xie J, Lin M, Geng Y, Li Y, Yuan J (2023). xgboost: Extreme Gradient

Boosting. R package version 1.7.6.1, <https://CRAN.R-project.org/package=xgboost>.

Greg R, Developers G (2024). gbm: Generalized Boosted Regression Models. R package version 2.1.9, <https://CRAN.Rproject.org/package=gbm>.

G. Ridgeway (1999). “The state of boosting,” Computing Science and Statistics 31:172-181.

Meyer D, Dimitriadou E, Hornik K, Weingessel A, Leisch F (2023). _e1071: Misc Functions of the Department of Statistics, Probability Theory Group (Formerly: E1071), TU Wien_. R package version 1.7-14, <https://CRAN.R-project.org/package=e1071>.

Liaw, A. and Wiener, M. (2002). Classification and Regression by randomForest. R News

2(3), 18--22.

Saglam F (2021). rbooster: AdaBoost Framework for Any Classifier. R package version 1.1. <https://CRAN.R-project.org/package=rbooster>.

**SupplementaryFile8. Cross-validated performance analysis of machine learning algorithms**.

Table S1. Repeated 5-fold cross-validation performance metrics of ten machine learning (SVM, RFF, MLR, ANN, XGB ADB, GBM, TRE, NBS, KNN) methods.

| Sl. No. | ML Method | Predictive accuracy |
| --- | --- | --- |
| 1 | SVM | 99.97 ± 0.042 |
| 2 | RFF | 99.98 ± 0.055 |
| 3 | MLR | 97.26 ± 0.084 |
| 4 | ANN | 94.84 ± 0.096 |
| 5 | XGB | 99.39 ± 0.079 |
| 6 | ADB | 99.75 ± 0.089 |
| 7 | GBM | 99.17 ± 0.078 |
| 8 | TRE | 84.22 ± 0.14 |
| 9 | NBS | 91.56 ± 0.095 |
| 10 | KNN | 97.77 ± 0.073 |

Values are shown in ($\mu\pm\sigma$) form, where, $\mu$: mean classification accuracy and $\sigma$: standard deviation of accuracy over the cross-validations; $\mu and \sigma$ values are averaged over the 500 repetitions.

**SupplementaryFile9. Independent evaluation of the ten trained serotype machine learning models on FMD virus isolates reported from the FMD-endemic countries including Kenya, Nepal, Bangladesh, Russia, Turkey, China, and Bhutan.**


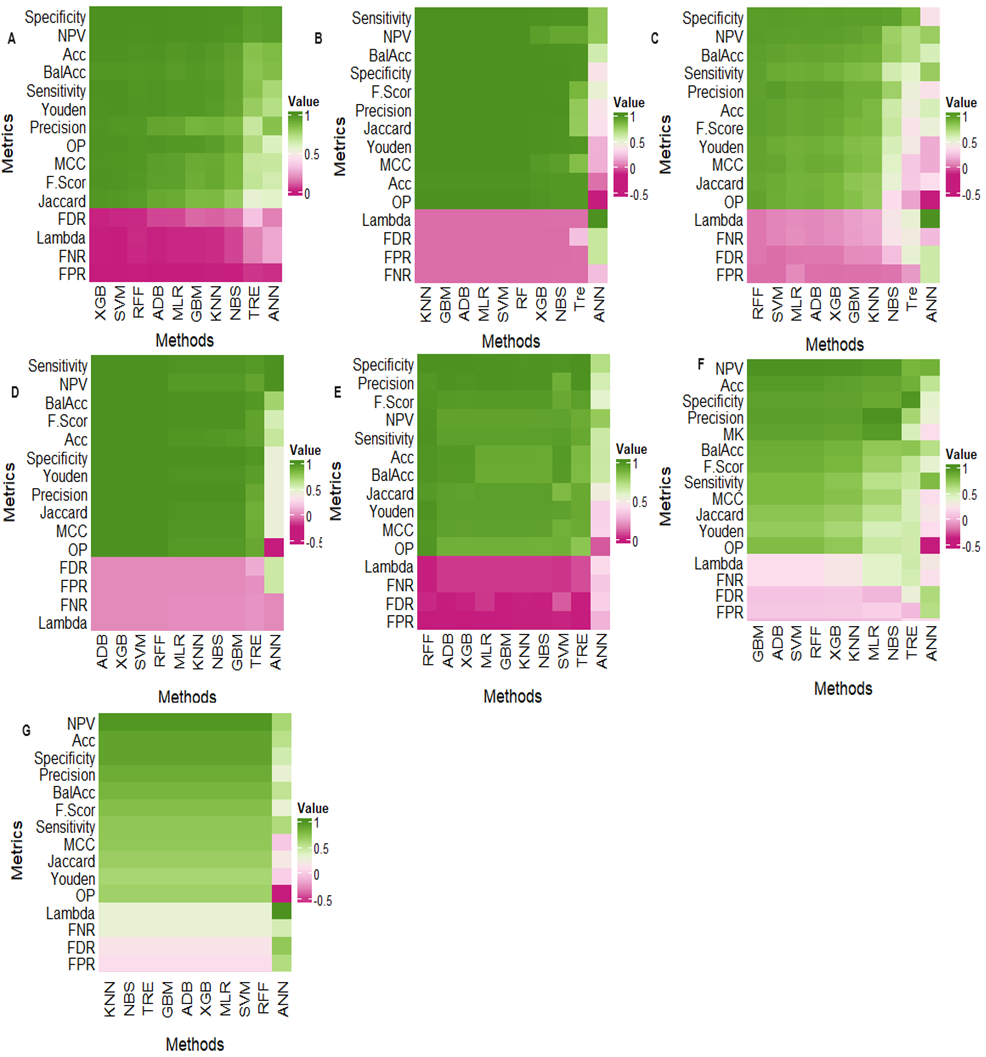


**Figure S1**. Performance analysis of machine learning algorithms on Independent datasets. The results are shown for (A) Kenya, (B) Nepal, (C) Bangladesh, (D) Russia, (E) Turkey, (F) China, and (G) Bhutan.

**SupplementaryFile10. Repeated five-fold cross-validation performance of four trained machine learning models (SVM, RFF, ADB, and XGB) for topotype prediction on FMD virus isolates.**

**
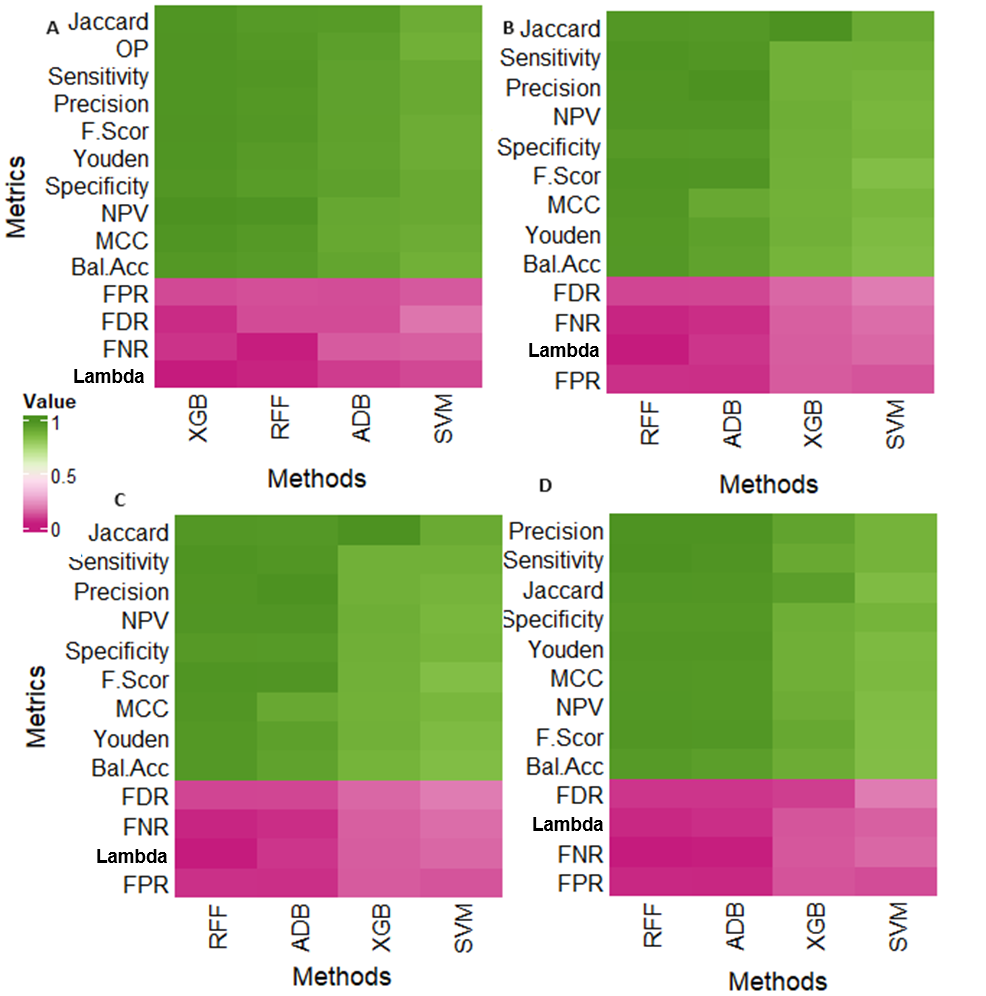
**

**Figure S2.** Performance of the top-four machine learning models (SVM, RFF, ADB, and XGB) for topotype prediction for four serotypes. The results are shown over 16 performance metrics for serotype C, SAT 1, SAT 2, and SAT 3.

**SupplementaryFile11. Utilization of the MolEpidPred approach for molecular epidemiology of field FMD virus isolates.**

The utility of the MolEpidPred prediction server was demonstrated on the 74 FMD field virus isolates reported from 45 FMD outbreak spots in India during the year 2028-19. The samples were collected from the state veterinary departments from cloven-hoofed animals including cattle, buffalo, pig, and goat and sent to the ICFMD lab, an FAO reference centre for FMD, of ICAR-NIFMD, Bhubaneswar. Here, the virus isolates are used to sequence the VP1 region of the FMD virus genome for further epidemiological studies using the procedure described in Supplementary File 4. Then, the VP1 nucleotide sequence of the 74 FMD field virus isolates were used in the MolEpidPred approach to predict various components of epidemiology (*i.e*., serotype, topotype, and lineage) of the FMD virus and results are shown in Table S1. Further, the results obtained from the MolEpidPred tool were compared with that of phylogenetic analysis through MEGA software (*ver*. 11).

**Table S1.** Prediction results of the MolEpidPred for FMD virus field isolates collected from various outbreak areas in India.

| **Isolate ID** | **Outbreak** | **Animal** | **SVM** | **RFF** | **ADB** | **XGB** | **Consensus** | **MEGA** |
| --- | --- | --- | --- | --- | --- | --- | --- | --- |
| IC02/2018 | Keonjhar, OD | Cattle | O/ME-SA/Ind2001 | O/ME-SA/Ind2001 | O/ME-SA/Ind2001 | O/ME-SA/Ind2001 | O/ME-SA/Ind2001 | O/ME-SA/Ind2001 |
| IC03/2018 | Keonjhar, OD | Cattle | O/ME-SA/Ind2001 | O/ME-SA/Ind2001 | O/ME-SA/Ind2001 | O/ME-SA/Ind2001 | O/ME-SA/Ind2001 | O/ME-SA/Ind2001 |
| IC14/2018 | Sambalpur, OD | Cattle | O/ME-SA/Ind2001 | O/ME-SA/Ind2001 | O/ME-SA/Ind2001 | O/ME-SA/Ind2001 | O/ME-SA/Ind2001 | O/ME-SA/Ind2001 |
| IC15/2018 | Sambalpur, OD | Cattle | O/ME-SA/Ind2001 | O/ME-SA/Ind2001 | O/ME-SA/Ind2001 | O/ME-SA/Ind2001 | O/ME-SA/Ind2001 | O/ME-SA/Ind2001 |
| IC16/2018 | Sindupur, OD | Cattle | O/ME-SA/Ind2001 | O/ME-SA/Ind2001 | O/ME-SA/Ind2001 | O/ME-SA/Ind2001 | O/ME-SA/Ind2001 | O/ME-SA/Ind2001 |
| IC18/2018 | Jabalpur, MP | Buffalo | O/ME-SA/Ind2001 | O/ME-SA/Ind2001 | O/ME-SA/Ind2001 | O/ME-SA/Ind2001 | O/ME-SA/Ind2001 | O/ME-SA/Ind2001 |
| IC19/2018 | Jabalpur, MP | Buffalo | O/ME-SA/Ind2001 | O/ME-SA/Ind2001 | O/ME-SA/Ind2001 | O/ME-SA/Ind2001 | O/ME-SA/Ind2001 | O/ME-SA/Ind2001 |
| IC20/2018 | Jabalpur, MP | Cattle | O/ME-SA/Ind2001 | O/ME-SA/Ind2001 | O/ME-SA/Ind2001 | O/ME-SA/Ind2001 | O/ME-SA/Ind2001 | O/ME-SA/Ind2001 |
| IC23/2018 | Jabalpur, MP | Cattle | O/ME-SA/Ind2001 | O/ME-SA/Ind2001 | O/ME-SA/Ind2001 | O/ME-SA/Ind2001 | O/ME-SA/Ind2001 | O/ME-SA/Ind2001 |
| IC67/2018 | Galshi, WB | Cattle | O/ME-SA/Ind2001 | O/ME-SA/Ind2001 | O/ME-SA/Ind2001 | O/ME-SA/Ind2001 | O/ME-SA/Ind2001 | O/ME-SA/Ind2001 |
| IC70/2018 | Medinapur, WB | Cattle | O/ME-SA/Ind2001 | O/ME-SA/Ind2001 | O/ME-SA/Ind2001 | O/ME-SA/Ind2001 | O/ME-SA/Ind2001 | O/ME-SA/Ind2001 |
| IC72/2018 | Medinapur, WB | Pig | O/ME-SA/Ind2001 | O/ME-SA/Ind2001 | O/ME-SA/Ind2001 | O/ME-SA/Ind2001 | O/ME-SA/Ind2001 | O/ME-SA/Ind2001 |
| IC117/2018 | Patna, Bihar | Cattle | O/ME-SA/Ind2001 | O/ME-SA/Ind2001 | O/ME-SA/Ind2001 | O/ME-SA/Ind2001 | O/ME-SA/Ind2001 | O/ME-SA/Ind2001 |
| IC119/2018 | Patna, Bihar | Cattle | O/ME-SA/Ind2001 | O/ME-SA/Ind2001 | O/ME-SA/Ind2001 | O/ME-SA/Ind2001 | O/ME-SA/Ind2001 | O/ME-SA/Ind2001 |
| IC120/2018 | Patna, Bihar | Cattle | O/ME-SA/Ind2001 | O/ME-SA/Ind2001 | O/ME-SA/Ind2001 | O/ME-SA/Ind2001 | O/ME-SA/Ind2001 | O/ME-SA/Ind2001 |
| IC128/2018 | Bhoipur, Bihar | Buffalo | O/ME-SA/Ind2001 | O/ME-SA/Ind2001 | O/ME-SA/Ind2001 | O/ME-SA/Ind2001 | O/ME-SA/Ind2001 | O/ME-SA/Ind2001 |
| IC132/2018 | Puraini, Bihar | Buffalo | O/ME-SA/Ind2001 | O/ME-SA/Ind2001 | O/ME-SA/Ind2001 | O/ME-SA/Ind2001 | O/ME-SA/Ind2001 | O/ME-SA/Ind2001 |
| IC166/2018 | CCARI, Goa | Pig | O/ME-SA/Ind2001 | O/ME-SA/Ind2001 | O/ME-SA/Ind2001 | O/ME-SA/Ind2001 | O/ME-SA/Ind2001 | O/ME-SA/Ind2001 |
| IC168/2018 | Raipur, CH | Cattle | O/ME-SA/Ind2001 | O/ME-SA/Ind2001 | O/ME-SA/Ind2001 | O/ME-SA/Ind2001 | O/ME-SA/Ind2001 | O/ME-SA/Ind2001 |
| IC169/2018 | Raipur, CH | Cattle | O/ME-SA/Ind2001 | O/ME-SA/Ind2001 | O/ME-SA/Ind2001 | O/ME-SA/Ind2001 | O/ME-SA/Ind2001 | O/ME-SA/Ind2001 |
| IC170/2018 | Raipur, CH | Cattle | O/ME-SA/Ind2001 | O/ME-SA/Ind2001 | O/ME-SA/Ind2001 | O/ME-SA/Ind2001 | O/ME-SA/Ind2001 | O/ME-SA/Ind2001 |
| IC202/2018 | Erode, TN | Cattle | O/ME-SA/SA2018 | O/ME-SA/SA2018 | O/ME-SA/SA2018 | O/ME-SA/SA2018 | O/ME-SA/SA2018 | O/ME-SA/SA2018 |
| IC207/2018 | Erode, TN | Cattle | O/ME-SA/SA2018 | O/ME-SA/SA2018 | O/ME-SA/SA2018 | O/ME-SA/SA2018 | O/ME-SA/SA2018 | O/ME-SA/SA2018 |
| IC214/2018 | Erode, TN | Cattle | O/ME-SA/Ind2001 | O/ME-SA/Ind2001 | O/ME-SA/Ind2001 | O/ME-SA/Ind2001 | O/ME-SA/Ind2001 | O/ME-SA/Ind2001 |
| IC218/2018 | Erode, TN | Cattle | O/ME-SA/SA2018 | O/ME-SA/SA2018 | O/ME-SA/SA2018 | O/ME-SA/SA2018 | O/ME-SA/SA2018 | O/ME-SA/SA2018 |
| IC221/2018 | Erode, TN | Cattle | O/ME-SA/SA2018 | O/ME-SA/SA2018 | O/ME-SA/SA2018 | O/ME-SA/SA2018 | O/ME-SA/SA2018 | O/ME-SA/SA2018 |
| IC231/2018 | Erode, TN | Cattle | O/ME-SA/SA2018 | O/ME-SA/SA2018 | O/ME-SA/SA2018 | O/ME-SA/SA2018 | O/ME-SA/SA2018 | O/ME-SA/SA2018 |
| IC233/2018 | Erode, TN | Cattle | O/ME-SA/SA2018 | O/ME-SA/SA2018 | O/ME-SA/SA2018 | O/ME-SA/SA2018 | O/ME-SA/SA2018 | O/ME-SA/SA2018 |
| IC454/2018 | Ramnagara, KN | Cattle | O/ME-SA/Ind2001 | O/ME-SA/Ind2001 | O/ME-SA/Ind2001 | O/ME-SA/Ind2001 | O/ME-SA/Ind2001 | O/ME-SA/SA2018 |
| IC491/2018 | Tumkur, KN | Cattle | O/ME-SA/Ind2001 | O/ME-SA/Ind2001 | O/ME-SA/Ind2001 | O/ME-SA/SA2018 | O/ME-SA/Ind2001 | O/ME-SA/Ind2001 |
| IC586/2018 | Chiballapur, KN | Cattle | O/ME-SA/Ind2001 | O/ME-SA/Ind2001 | O/ME-SA/Ind2001 | O/ME-SA/Ind2001 | O/ME-SA/Ind2001 | O/ME-SA/Ind2001 |
| IC4/2019 | Dibrugarh, AS | Cattle | O/ME-SA/Ind2001 | O/ME-SA/Ind2001 | O/ME-SA/Ind2001 | O/ME-SA/Ind2001 | O/ME-SA/Ind2001 | O/ME-SA/Ind2001 |
| IC11/2019 | Darrang, AS | Cattle | O/ME-SA/Ind2001 | O/ME-SA/Ind2001 | O/ME-SA/Ind2001 | O/ME-SA/Ind2001 | O/ME-SA/Ind2001 | O/ME-SA/Ind2001 |
| IC15/2019 | Debipur, TR | Cattle | O/ME-SA/Ind2001 | O/ME-SA/Ind2001 | O/ME-SA/Ind2001 | O/ME-SA/Ind2001 | O/ME-SA/Ind2001 | O/ME-SA/Ind2001 |
| IC19/2019 | Gurdaspur, PB | Cattle | O/ME-SA/Ind2001 | O/ME-SA/Ind2001 | O/ME-SA/Ind2001 | O/ME-SA/Ind2001 | O/ME-SA/Ind2001 | O/ME-SA/Ind2001 |
| IC20/2019 | Gurdaspur, PB | Cattle | O/ME-SA/Ind2001 | O/ME-SA/Ind2001 | O/ME-SA/Ind2001 | O/ME-SA/Ind2001 | O/ME-SA/Ind2001 | O/ME-SA/Ind2001 |
| IC23/2019 | Chamker S, PB | Cattle | O/ME-SA/Ind2001 | O/ME-SA/Ind2001 | O/ME-SA/Ind2001 | O/ME-SA/Ind2001 | O/ME-SA/Ind2001 | O/ME-SA/Ind2001 |
| IC28/2019 | Dhel, MP | Cattle | O/ME-SA/Ind2001 | O/ME-SA/Ind2001 | O/ME-SA/Ind2001 | O/ME-SA/SA2018 | O/ME-SA/Ind2001 | O/ME-SA/Ind2001 |
| IC30/2019 | Dhel, MP | Cattle | O/ME-SA/Ind2001 | O/ME-SA/SA2018 | O/ME-SA/SA2018 | O/ME-SA/SA2018 | O/ME-SA/SA2018 | O/ME-SA/Ind2001 |
| IC31/2019 | Dhel, MP | Cattle | O/ME-SA/Ind2001 | O/ME-SA/Ind2001 | O/ME-SA/Ind2001 | O/ME-SA/SA2018 | O/ME-SA/Ind2001 | O/ME-SA/Ind2001 |
| IC21/2019 | Gurdaspur, PB | Cattle | O/ME-SA/Ind2001 | O/ME-SA/Ind2001 | O/ME-SA/Ind2001 | O/ME-SA/Ind2001 | O/ME-SA/Ind2001 | O/ME-SA/Ind2001 |
| IC22/2019 | Gurdaspur, PB | Cattle | O/ME-SA/Ind2001 | O/ME-SA/Ind2001 | O/ME-SA/Ind2001 | O/ME-SA/Ind2001 | O/ME-SA/Ind2001 | O/ME-SA/Ind2001 |
| IC27/2019 | Dhel, MP | Cattle | O/ME-SA/Ind2001 | O/ME-SA/SA2018 | O/ME-SA/SA2018 | O/ME-SA/SA2018 | O/ME-SA/SA2018 | O/ME-SA/Ind2001 |
| IC43/2019 | Ahemed N, MH | Cattle | O/ME-SA/Ind2001 | O/ME-SA/Ind2001 | O/ME-SA/Ind2001 | O/ME-SA/Ind2001 | O/ME-SA/Ind2001 | O/ME-SA/Ind2001 |
| IC44/2019 | Ahemed N, MH | Cattle | O/ME-SA/Ind2001 | O/ME-SA/Ind2001 | O/ME-SA/Ind2001 | O/ME-SA/Ind2001 | O/ME-SA/Ind2001 | O/ME-SA/Ind2001 |
| IC45/2019 | Ahemed N, MH | Cattle | O/ME-SA/Ind2001 | O/ME-SA/Ind2001 | O/ME-SA/Ind2001 | O/ME-SA/Ind2001 | O/ME-SA/Ind2001 | O/ME-SA/Ind2001 |
| IC6/2019 | Mangaldai, AS | Cattle | Asia 1/ASIA/C | Asia 1/ASIA/C | Asia 1/ASIA/C | Asia 1/ASIA/C | Asia 1/ASIA/C | NA |
| IC51/2019 | SAS Nagar, PB | Cattle | O/ME-SA/Ind2001 | O/ME-SA/Ind2001 | O/ME-SA/Ind2001 | O/ME-SA/Ind2001 | O/ME-SA/Ind2001 | O/ME-SA/Ind2001 |
| IC52/2019 | SAS Nagar, PB | Cattle | O/ME-SA/Ind2001 | O/ME-SA/Ind2001 | O/ME-SA/Ind2001 | O/ME-SA/Ind2001 | O/ME-SA/Ind2001 | O/ME-SA/Ind2001 |
| IC56/2019 | Tarn Taran, PB | Cattle | O/ME-SA/Ind2001 | O/ME-SA/Ind2001 | O/ME-SA/Ind2001 | O/ME-SA/Ind2001 | O/ME-SA/Ind2001 | O/ME-SA/Ind2001 |
| IC690/2018 | Udaipur, RJ | Cattle | O/ME-SA/Ind2001 | O/ME-SA/Ind2001 | O/ME-SA/Ind2001 | O/ME-SA/Ind2001 | O/ME-SA/Ind2001 | O/ME-SA/Ind2001 |
| IC705/2018 | Jaipur, RJ | Cattle | O/ME-SA/Ind2001 | O/ME-SA/Ind2001 | O/ME-SA/Ind2001 | O/ME-SA/Ind2001 | O/ME-SA/Ind2001 | O/ME-SA/Ind2001 |
| IC706/2018 | Jaipur, RJ | Cattle | O/ME-SA/Ind2001 | O/ME-SA/Ind2001 | O/ME-SA/Ind2001 | O/ME-SA/Ind2001 | O/ME-SA/Ind2001 | O/ME-SA/Ind2001 |
| IC721/2018 | Beed, MH | Goat | O/ME-SA/SA2018 | O/ME-SA/SA2018 | O/ME-SA/Ind2001 | O/ME-SA/SA2018 | O/ME-SA/SA2018 | O/ME-SA/SA2018 |
| IC722/2018 | Beed, MH | Goat | O/ME-SA/SA2018 | O/ME-SA/SA2018 | O/ME-SA/Ind2001 | O/ME-SA/SA2018 | O/ME-SA/SA2018 | O/ME-SA/SA2018 |
| IC59/2019 | TaranTaran, PB | Cattle | O/ME-SA/Ind2001 | O/ME-SA/Ind2001 | O/ME-SA/Ind2001 | O/ME-SA/Ind2001 | O/ME-SA/Ind2001 | O/ME-SA/Ind2001 |
| IC94/2019 | Gondia, MH | Cattle | O/ME-SA/Ind2001 | O/ME-SA/Ind2001 | O/ME-SA/Ind2001 | O/ME-SA/Ind2001 | O/ME-SA/Ind2001 | O/ME-SA/Ind2001 |
| IC130/2019 | Pune, MH | Cattle | O/ME-SA/SA2018 | O/ME-SA/SA2018 | O/ME-SA/Ind2001 | O/ME-SA/SA2018 | O/ME-SA/SA2018 | O/ME-SA/SA2018 |
| IC133/2019 | Pune, MH | Cattle | O/ME-SA/SA2018 | O/ME-SA/SA2018 | O/ME-SA/Ind2001 | O/ME-SA/SA2018 | O/ME-SA/SA2018 | O/ME-SA/SA2018 |
| IC141/2019 | Hissar, HR | Cattle | O/ME-SA/Ind2001 | O/ME-SA/Ind2001 | O/ME-SA/Ind2001 | O/ME-SA/Ind2001 | O/ME-SA/Ind2001 | O/ME-SA/Ind2001 |
| IC452/2018 | Kolar, KN | Cattle | O/ME-SA/Ind2001 | O/ME-SA/Ind2001 | O/ME-SA/Ind2001 | O/ME-SA/Ind2001 | O/ME-SA/Ind2001 | O/ME-SA/Ind2001 |
| IC453/2018 | Ramanagara, KN | Cattle | O/ME-SA/Ind2001 | O/ME-SA/Ind2001 | O/ME-SA/Ind2001 | O/ME-SA/Ind2001 | O/ME-SA/Ind2001 | O/ME-SA/Ind2001 |
| IC467/2018 | Bangaluru (R), KN | Cattle | O/ME-SA/Ind2001 | O/ME-SA/Ind2001 | O/ME-SA/Ind2001 | O/ME-SA/Ind2001 | O/ME-SA/Ind2001 | O/ME-SA/Ind2001 |
| IC627/2018 | Tumakuru, KN | Cattle | O/ME-SA/SA2018 | O/ME-SA/SA2018 | O/ME-SA/Ind2001 | O/ME-SA/SA2018 | O/ME-SA/SA2018 | O/ME-SA/SA2018 |
| IC741/2018 | Puducherry | Cattle | O/ME-SA/Ind2001 | O/ME-SA/Ind2001 | O/ME-SA/Ind2001 | O/ME-SA/Ind2001 | O/ME-SA/Ind2001 | O/ME-SA/Ind2001 |
| IC123/2019 | Nadia, WB | Cattle | O/ME-SA/Ind2001 | O/ME-SA/Ind2001 | O/ME-SA/Ind2001 | O/ME-SA/Ind2001 | O/ME-SA/Ind2001 | O/ME-SA/Ind2001 |
| IC124/2019 | Bankara, WB | Cattle | O/ME-SA/Ind2001 | O/ME-SA/Ind2001 | O/ME-SA/Ind2001 | O/ME-SA/Ind2001 | O/ME-SA/Ind2001 | O/ME-SA/Ind2001 |
| IC142/2019 | Hisar, HR | Buffalo | O/ME-SA/Ind2001 | O/ME-SA/Ind2001 | O/ME-SA/Ind2001 | O/ME-SA/Ind2001 | O/ME-SA/Ind2001 | O/ME-SA/Ind2001 |
| IC419/2018 | Bengaluru (S), KN | Cattle | O/ME-SA/SA2018 | O/ME-SA/SA2018 | O/ME-SA/Ind2001 | O/ME-SA/SA2018 | O/ME-SA/SA2018 | O/ME-SA/SA2018 |
| IC437/2018 | Bengaluru (U), KN | Cattle | O/ME-SA/Ind2001 | O/ME-SA/Ind2001 | O/ME-SA/Ind2001 | O/ME-SA/Ind2001 | O/ME-SA/Ind2001 | O/ME-SA/Ind2001 |
| IC441/2018 | Bengaluru (N), KN | Cattle | O/ME-SA/Ind2001 | O/ME-SA/Ind2001 | O/ME-SA/Ind2001 | O/ME-SA/Ind2001 | O/ME-SA/Ind2001 | O/ME-SA/Ind2001 |
| IC540/2018 | Raichur, KN | Cattle | O/ME-SA/Ind2001 | O/ME-SA/Ind2001 | O/ME-SA/Ind2001 | O/ME-SA/Ind2001 | O/ME-SA/Ind2001 | O/ME-SA/Ind2001 |
| IC613/2018 | Dharwada, KN | Cattle | O/ME-SA/SA2018 | O/ME-SA/SA2018 | O/ME-SA/Ind2001 | O/ME-SA/SA2018 | O/ME-SA/SA2018 | O/ME-SA/SA2018 |
| IC614/2018 | Dharwada, KN | Cattle | O/ME-SA/SA2018 | O/ME-SA/SA2018 | O/ME-SA/Ind2001 | O/ME-SA/SA2018 | O/ME-SA/SA2018 | O/ME-SA/SA2018 |

OD: Odisha; MP: Madhya Pradesh; KN: Karnataka; TN: Tamil Nadu; RJ: Rajasthan; HR: Haryana; MH: Maharashtra; PB: Punjab; WB: West Bengal; CH: Chhattisgarh; AS: Assam; TR: Tripura; Dhel: Dhelekhedivisha

**SupplementaryFile12. MolEpidPred prediction server available at** [**https://nifmd-bbf.icar.gov.in/MolEpidPred**](https://nifmd-bbf.icar.gov.in/MolEpidPred) **for molecular epidemiology of the FMD virus isolates.**


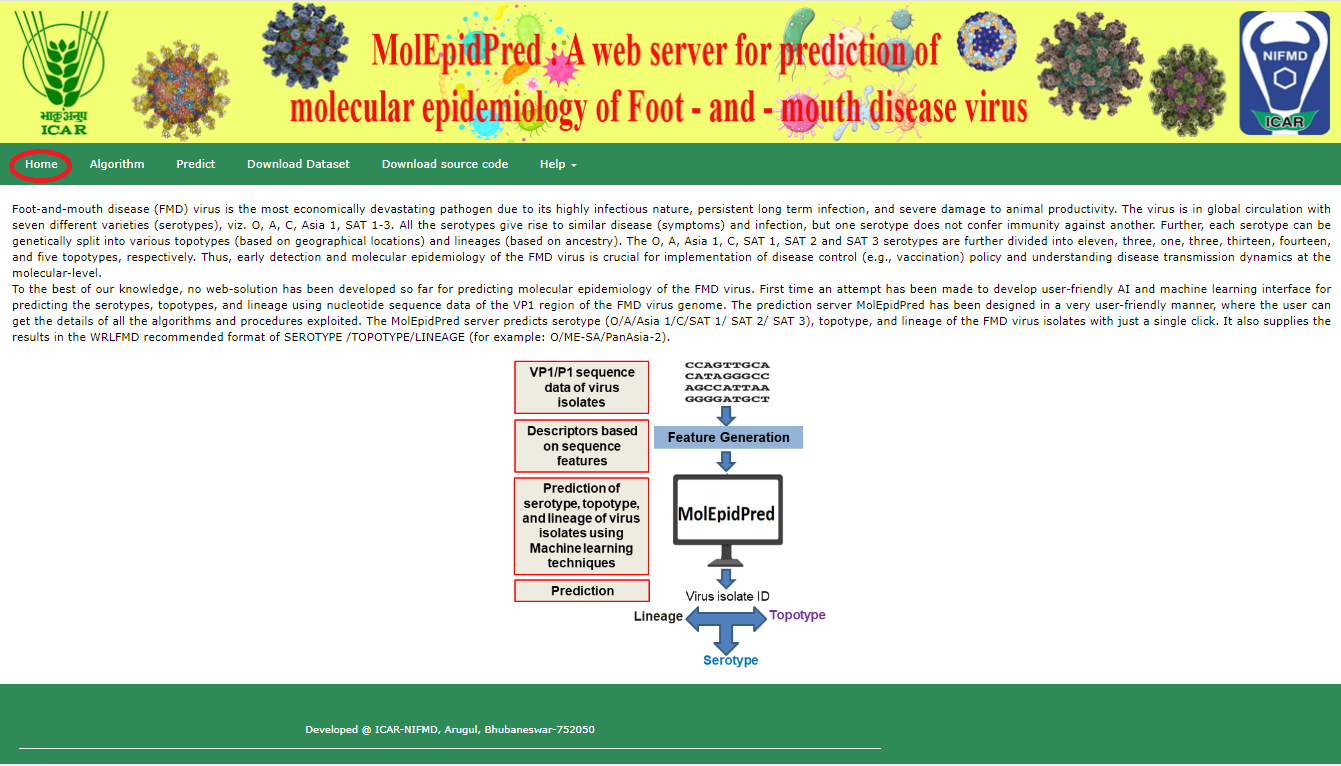


**Figure S1. Homepage of the MolEpidPred web prediction server.**


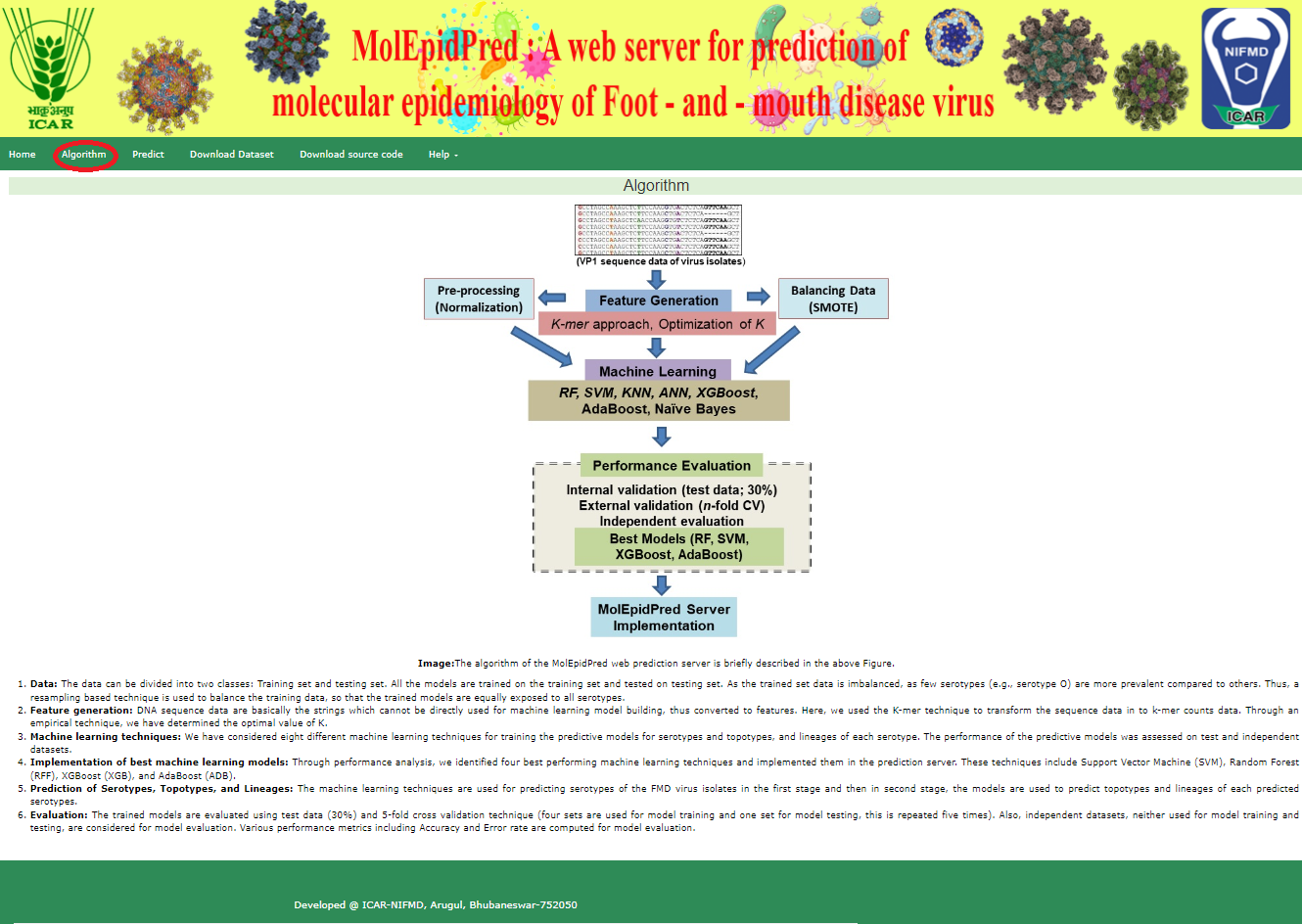


**Figure S2**. **Algorithm page of the MolEpidPred web prediction server.**

**
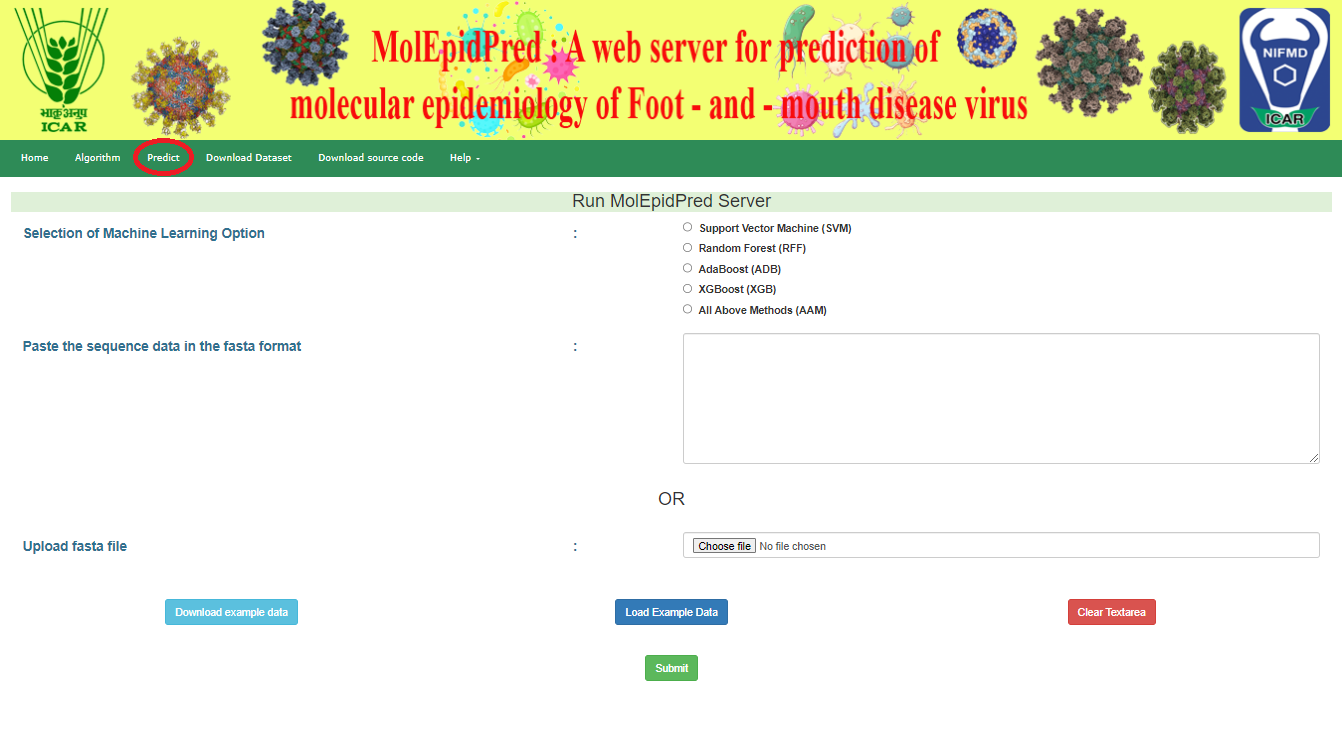
Figure S3.** **Execution page of the MolEpidPred web prediction server.**

**Figure S4. Output/result page of the MolEpidPred web prediction server.**


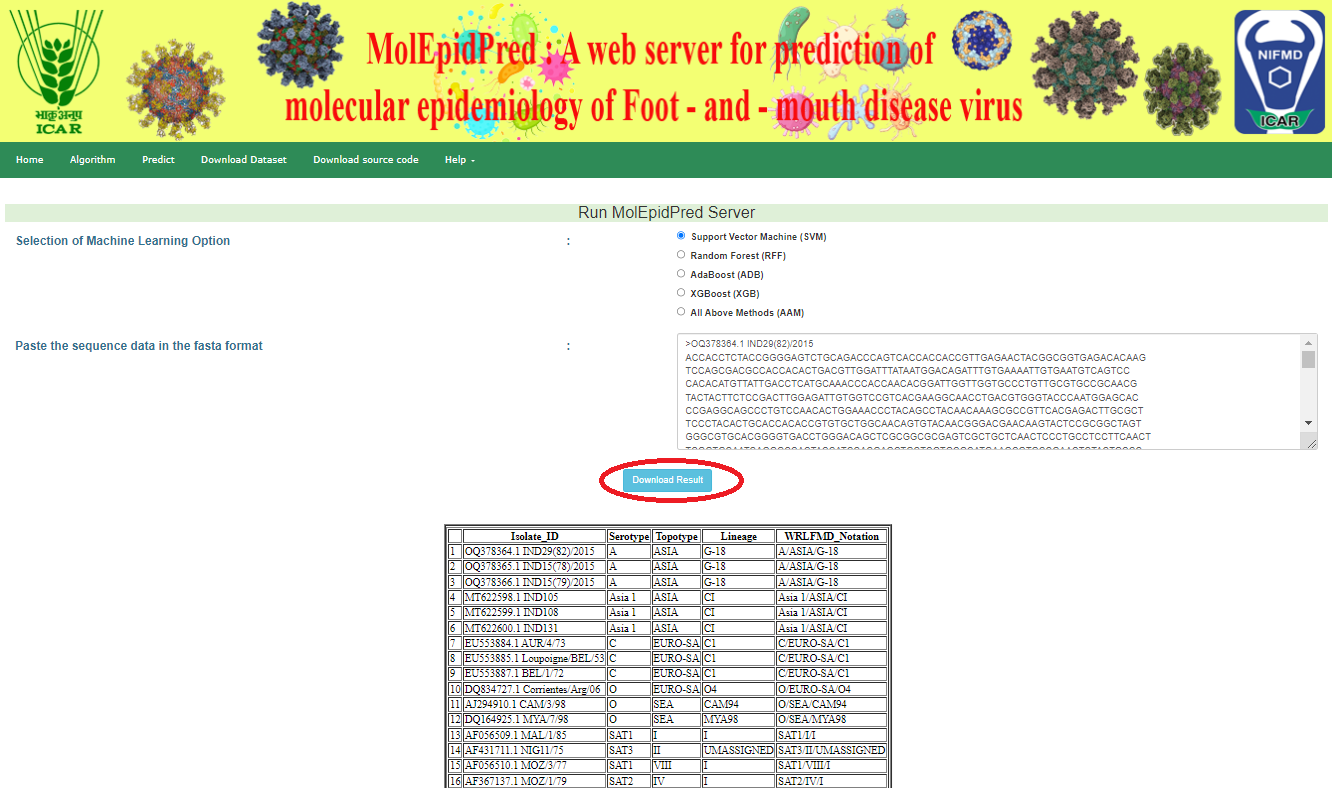


**Figure S5. Manual/Help page of the MolEpidPred web prediction server.**


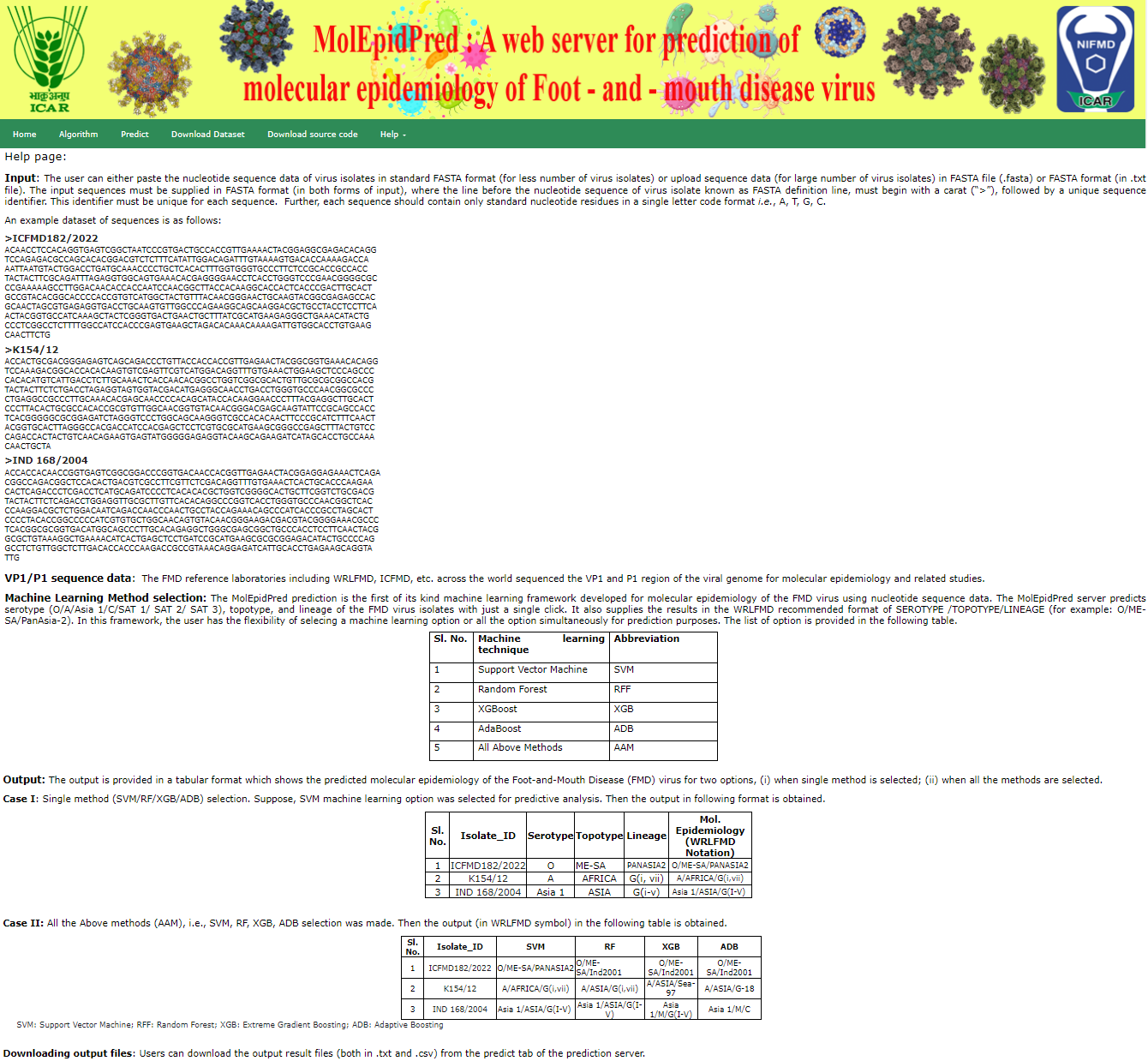

Supplement: Supplementary_Materials_elaf001 [file supplementary_materials_elaf001.docx]
